# Combining fecal microbiome and metabolomics to reveal the disturbance of gut microbiota in liver injury and the therapeutic mechanism of shaoyao gancao decoction

**DOI:** 10.3389/fphar.2022.911356

**Published:** 2022-08-16

**Authors:** Jingwei Li, Min Zhao, Jianming Li, Miao Wang, Chunjie Zhao

**Affiliations:** ^1^ School of Pharmacy, Shenyang Pharmaceutical University, Shenyang, China; ^2^ Guangxi University of Chinese Medicine, Nanning, China; ^3^ School of Life Science and Biopharmaceutics, Shenyang Pharmaceutical University, Shenyang, China

**Keywords:** liver injury, shaoyao gancao decoction, gut microbiota, fecal metabolomics, short-chain fatty acids, correlation analysis

## Abstract

Chemical liver injury is closely related to gut microbiota and its metabolites. In this study, we combined 16S rRNA gene sequencing, ^1^H NMR-based fecal metabolomics and GC-MS to evaluate the changes in gut microbiota, fecal metabolites and Short-chain fatty acids (SCFAs) in CCl_4_-induced liver injury in Sprague-Dawley rats, and the therapeutic effect of Shaoyao Gancao Decoction (SGD). The results showed that CCl_4_-induced liver injury overexpressed CYP2E1, enhanced oxidative stress, decreased antioxidant enzymes (SOD, GSH), increased peroxidative products MDA and inflammatory responses (IL-6, TNF-α), which were ameliorated by SGD treatment. H&E staining showed that SGD could alleviate liver tissue lesions, which was confirmed by the recovered liver index, ALT and AST. Correlation network analysis indicated that liver injury led to a decrease in microbiota correlation, while SGD helped restore it. In addition, fecal metabolomic confirmed the PICRUSt results that liver injury caused disturbances in amino acid metabolism, which were modulated by SGD. Spearman’s analysis showed that liver injury disrupted ammonia transport, urea cycle, intestinal barrier and energy metabolism. Moreover, the levels of SCFAs were also decreased, and the abundance of Lachnoclostridium, Blautia, Lachnospiraceae_NK4A136_group, UCG-005 and Turicibacter associated with SCFAs were altered. However, all this can be alleviated by SGD. More importantly, pseudo germ-free rats demonstrated that the absence of gut microbiota aggravated liver injury and affected the efficacy of SGD. Taken together, we speculate that the gut microbiota has a protective role in the pathogenesis of liver injury, and has a positive significance for the efficacy of SGD. Moreover, SGD can treat liver injury by modulating gut microbiota and its metabolites and SCFAs. This provides useful evidence for the study of the pathogenesis of liver injury and the clinical application of SGD.

## 1 Introduction

The liver, as the organ to which exogenous substances were initially contacted, was most vulnerable to chemical induction injury. Chemical liver injury refers to the liver injury caused by chemical hepatotoxic substances such as alcohol, drugs, heavy metals, and chemically toxic substances (Gu and Manautou, 2012). As a typical environmental toxicant, CCl_4_ is widely used to induce experimental liver disease in animal models (Unsal et al., 2021). Toxins are metabolized *in vivo* by cytochrome P450 enzymes into harmful reactive intermediates, which can generate oxidative stress. It further causes the dysregulation of cell signaling pathways and the dysfunction of biological macromolecules, resulting in cell damage (Rahman et al., 2021). Liver injury activates myofibroblasts, which secrete large amounts of extracellular matrix, resulting in nonspecific changes in liver structure and function, and ultimately developing into liver fibrosis (Campana and Iredale, 2017). However, as an acquired disease, it can be prevented (Michalopoulos, 2017).

The liver and the intestine share a common embryological origin, and the two have extensive structural and functional connections, so the theory of the gut-liver axis is proposed. The gut-liver axis refers to the bidirectional relationship established between the gut, microbiota, and liver through the portal vein (Albillos et al., 2020). Products derived from the gut are transported through the portal vein to the liver, which in turn affects gut function through bile secretion and enterohepatic circulation. Numerous studies have shown that dysbiosis in the gut microbiota is associated with liver disease (Kong et al., 2021). In a state of liver injury, decreased small intestinal motility, abnormal bile acid secretion, increased intestinal mucosal permeability, and impaired intestinal immunity lead to altered gut microbiota and bacterial overgrowth. Moreover, it leads to the increase of harmful substances in the systemic circulation and promotes inflammation (Tilg et al., 2020). Furthermore, bacterial metabolites are associated with the development and treatment of liver disease (Levy et al., 2017). SCFAs are the metabolites of gut microbes, which can provide energy to intestinal epithelial cells, maintain the balance of water and electrolytes, regulate the balance of gut microbiota, anti-inflammatory and regulate gene expression (Martin-Gallausiaux et al., 2021). Therefore, the gut-liver axis theory provides a new idea for the study of the treatment mechanism of liver diseases. At present, the research on the treatment of liver injury by improving the gut microbiota has attracted more and more attention (Wang et al., 2021; Li et al., 2022).

SGD is a famous traditional Chinese medicine prescription from Treatise on Febrile Diseases. It is composed of Paeonia lactiflora Pall [Paeoniaceae; Paeoniae Radix Alba] and *Glycyrrhiza* glabra L [Fabaceae; Glycyrrhizae Radix et Rhizoma Praeparata Cum Melle]. Modern pharmacology and clinical practice show that SGD can be used to treat liver and spleen disorders (Bi et al., 2014). In previous studies, we have characterized the chemical profiles of SGD. 73 compounds were identified, including phenols and monoterpenoids, triterpenoid saponins and flavonoids. It laid a material basis for the pharmacodynamic study of SGD (Sun et al., 2020a). In addition, we also studied the mechanism of SGD in the treatment of liver injury from the perspective of metabolomics. By ^1^H NMR and UPLC-MS, 26 important metabolites were identified, mainly involved in amino acid and lipid metabolism, revealing the metabolic mechanism of SGD in the treatment of liver injury (Sun et al., 2020b).

Studies have shown that there is an interaction between gut microbiota and botanical drugs. The gut microbiota can alter the chemical structure and biological activity of drugs through enzymatic reactions, and botanical drugs modulate the composition and structure of the gut microbiota and its secretions, thereby improving metabolic disorders (Arquero Avilès et al., 2014; An et al., 2019). However, the mechanism of SGD in the treatment of liver injury based on the gut-liver axis theory is less reported. Therefore, we assessed disturbing changes in gut microbiota by 16S rRNA gene sequencing and analyzed changes in fecal metabolites and SCFAs by ^1^H NMR-based fecal metabolomics and GC-MS. In addition, the effect of gut microbiota on the therapeutic effect of SGD was analyzed by establishing a pseudo germ-free (PGF) rats model. Reveal the mechanism of SGD in the treatment of liver injury from the perspective of gut microbiota. It provides a new theoretical basis for the clinical application of SGD in the treatment of liver injury.

## 2 Materials and methods

### 2.1 Drugs and chemicals

Paeonia lactiflora Pall [Paeoniaceae; Paeoniae Radix Alba] (Baishao, batch number: 18061201; source: Anhui China), *Glycyrrhiza* glabra L [Fabaceae; Glycyrrhizae Radix et Rhizoma Praeparata Cum Melle] (Zhigancao, batch number: 180518; source: Neimenggu China) were collected from Guoda Pharmacy (Shenyang, China).

Cefadroxil, neomycin sulfate and erythromycin were purchased from Shanghai yuan ye Bio-Technology Co., Ltd (Shanghai, China). Cytochrome P450 2E1 (CYP2E1), Interleukin 6 (IL-6) and Tumor necrosis factorα (TNF-α) ELISA Kit instructions were provided by Jiangsu Baolai Biotechnology Co., Ltd (Jiangsu, China). Dibasic sodium phosphate and sodium dihydrogen phosphate were purchased from Kemeo Regent Co., Ltd (Tianjin, China). Sodium 3trimethylsilyl-propionate [2,2,3,3,d4] (TSP) and deuteroxide (D_2_O) were obtained from Merck Drugs and Biotechnology (Darmstadt, Germany). Purified water was supplied by Wahaha Co., Ltd (Hangzhou, China). Acetic acid, propionic acid, isobutyric acid, butyric acid, isovaleric acid, valeric acid, caproic acid and crotonic acid were purchased from Shanghai Macklin Biochemical Co., Ltd (Shanghai, China). Propyl chloroformate (PCF) was purchased from Shanghai Yien Chemical Technology Co., Ltd (Shanghai, China). Sodium hydroxide and pyridine were provided by Tianjin Hengxing Chemical Preparation Co., Ltd (Tianjin, China). n-Propanol was purchased from Damao Chemical Reagent Factory (Tianjin, China). n-Hexane was bought in Tianjin Fuyu Fine Chemical Co., Ltd (Tianjin, China).

### 2.2 Animals

Thirty male Sprague-Dawley (SD) rats (10 weeks, 200 ± 20 g) purchased from the Experimental Animal Center of Shenyang Pharmaceutical University (Shenyang, China) were placed in a 12-h light-dark cycle with a temperature of 22 ± 2 C and relative humidity of 50 ± 10%. Free access to food and water during the adaptation period of 1 week before the experiment.

### 2.3 Preparation of SGD

250 g of Paeonia lactiflora Pall [Paeoniaceae; Paeoniae Radix Alba] and 250 g of *Glycyrrhiza* glabra L [Fabaceae; Glycyrrhizae Radix et Rhizoma Praeparata Cum Melle] were macerated in 5 L of purified water for 0.5 h. After decocting for 1.5 h, the extract was filtered with 5-layer of gauzes. Add 1:8 (W/V) water to the drug residue, and repeat the above steps twice, each time decocting for 1 h. These filtrates were mixed and freeze-dried. A brown powder was obtained, and the extraction rate of SGD was 33.35%.

### 2.4 Animals treatment

Pseudo germ-free (PGF) rats were established by giving broad-spectrum antibiotics to explore whether SGD can treat the liver injury by regulating gut microbiotas. Broad-spectrum antibiotics contain cefadroxil (100 mg/kg), neomycin sulfate (300 mg/kg) and erythromycin (300 mg/kg) (Zhang et al., 2021a).

All rats were randomly divided into five groups (n = 6): control group (Con), liver injury model group (LIM), SGD treatment group (SGDT), pseudo germ-free liver injury model group (PLIM) and pseudo germ-free SGD treatment group (PSGDT). The liver injury model was established by gavage of CCl_4_. LIM, SGDT, PLIM and PSGDT were given 40% CCl_4_ soybean oil solution by gavage (first dose of 5 ml/kg followed by 3 ml/kg) twice a week for 8 weeks. Con was given the same amount of soybean oil. From the third week, SGDT and PSGDT were given SGD (1.5 g/kg) orally once a day (Sun et al., 2020b). At the same time, PLIM and PSGDT were given antibiotics (twice a day for 3 days before administration and once a day after administration). Meanwhile, other groups were given the same amount of water. Record the weight of rats every week (Zhang et al., 2021a).

### 2.5 Sample collections and preparation

At the end of the eighth week, fecal samples were collected continuously for 3 days in the absence of specific pathogens (SPF) using sterile metabolic cages for metabolomics analysis. After the last administration, disinfect the perianal and caudal parts of rats with 75% alcohol cotton on a laminar flow workbench under sterile conditions, and the fecal samples were collected by tail lifting method with the aseptic freezing tube for sequencing analysis of 16S rRNA gene. All stool samples mentioned above were stored at -80 C.

At the second, fourth, sixth and eighth weeks, the blood of the orbital venous plexus was collected and transferred to an EP tube. After standing at 4 C for 2 h, centrifuge at 4,000 rpm for 10 min to obtain serum samples for biochemical analysis. Fasting for 12 h after the last administration, anesthesia with 10% chloral hydrate, collecting blood from abdominal aorta into vacuum blood collection tube containing heparin. A plasma sample was obtained by centrifugation at 4,000 rpm for 10 min. The liver, spleen and thymus of each rat were taken out and washed with precooled normal saline. After drying, weighing, and calculating organ index according to the percentage of organs to body weight. The liver was divided into two parts, one of which was fixed in 10% formaldehyde for histology. In the other part, 1 g of the liver was put into a centrifuge tube, 9 ml of precooled normal saline was added, and it was prepared in a tissue homogenizer. After centrifugation at 4,000 rpm for 10 min, the supernatant was collected to obtain liver homogenate, which was stored at -80 C.

### 2.6 Histological analysis

After the liver was fixed with 10% formaldehyde for 48 h, it was embedded in paraffin. Tissue sections were stained with hematoxylin and eosin (H&E), and histopathological changes were observed under an optical microscope.

### 2.7 Biochemical assays

Serum liver function indexes, including alanine aminotransferase (ALT), aspartate aminotransferase (AST), albumin (ALB) and total protein (TP), were determined by an automatic biochemical analyzer BX-3010BX-3010 (Sysmex Ltd., Kobe, Japan) in the General Hospital of the Northern Theater of the Chinese People’s Liberation Army.

The levels of antioxidant indexes, malondialdehyde (MDA), superoxide dismutase (SOD) and glutathione (GSH), in liver homogenate were detected by using the kit produced by Nanjing Jiancheng Bioengineering Institute according to the instructions. The content of protein was determined by the BCA protein assay kit.

The level of CYP2E1 in liver homogenate was determined by ELISA kit according to the manufacturer’s instructions.

### 2.8 Measurement of inflammatory parameters in plasma

According to the manufacturer’s instructions, the levels of IL-6 and TNF-α in plasma were measured by ELISA kit.

### 2.9 16S rRNA gene sequencing analysis

The fecal samples were sequenced by 16S rRNA gene in Shanghai Major Biobio-pharm Technology Co., Ltd (Shanghai, China). Briefly, on ABI Gene AMP9700 (ABI, CA, USA), the V3V4 hypervariable regions of bacterial 16S rRNA gene were amplified by Transgen AP221-02: Transstart fast pfu DNA polymerase with primers of 338F (5′-ACT​CCT​ACG​GGA​GGC​AGC​AG -3′) and 806R (5′-GGACTACHVGGGTWTCTAAT -3′). Then, the PCR products were quantified by QuantiFluor™ -ST (Promega, USA). Finally, according to the existing protocols, the paired-end sequencing was carried out on the Illumina MiSeq platform (Illumina, San Diego, USA).

The original reading of the 16S rRNA gene sequence was spliced by Flash (version 1.2.11). Sequences were identified and filtered by UPARSE (Version 7.1 http://drive5.com/uparse/) in Qiime (version 1.9.1), and non-repetitive sequences (excluding single sequences) were clustered by OTU according to 97% similarity. Then, using RDP Classifier (Version 2.2 http://sourceforge.net/projects/rdp-classifier/) Bayesian algorithm, the representative sequences of OTU with 97% similarity level were taxonomically analyzed at the confidence threshold of 0.7. Moreover, the community species composition was counted at Phylum, Class, Order, Family and Genu levels.

Bioinformatics analysis was performed on the Majorbio Cloud Platform (http://www.majorbio.com/). Chao, Shannoneven, Shannon, Simpson and coverage index were used to evaluate the alpha diversity in the sample. Unweighted Pair-group Method with Arithmetic Mean (UPGMA), Principal Component Analysis (PCA), Principal co-ordinates analysis (PCoA) and Non-metric multidimensional scaling analysis (NMDS) were used to analyze beta diversity among groups, so as to study the difference of community structure among different samples. ANOSIM and PERMANOVA analysis were used to determine whether grouping was meaningful. R (version 3.3.1)' s stats package and python’s scipy package were used to identify the species with significant differences at various levels. Furthermore, the linear discriminant analysis (LDA) effect size (LEfSe) analysis was used to identify the key bacterial genera with significant differences in the experimental group. The value of LDA > 2 and P < 0.05 were considered as a significantly enriched community. db-RDA was carried out in Vegan package of R language (Version 3.3.1). NetworkX was used to explore the relationship among the top 50 microbial communities in genus. Correlations between gut microbiota were calculated by Spearman, and the absolute value of correlation coefficient was ≥0.5, P < 0.05. Transitivity, degree centrality (DC), closeness centrality (CC) and betweenness centrality (BC) were used to describe the topological characteristics of networks. Use PICRUSt (version 2.2.0) and Kyoto Encyclopedia of Genes and Genomes (KEGG) to predict the function of microbial community. Import the obtained KEGG-Pathway-Level 2 into STAMP (version 2.1.3), and remove the unclassified reads to determine the metabolic categories with significant differences.

### 2.10 Fecal metabolomics analysis

Fecal metabolomics was analyzed by ^1^H NMR. The feces were thawed at 4°C and ground into powder by mortar. 100 mg fecal sample was added into 1 ml precooled PBS (NaH_2_PO_4_-Na_2_HPO_4_, 0.2 M, pH 7.4) and vortexed for 30 s 13000 rpm for 10 min after 30min of ultrasound. Add 450 μl supernatant into 100 μl TSP D_2_O solution (1.0 mg/ml), vortex for 30 s, and transfer to 5 mm inner diameter nuclear magnetic tube.

Analysis by ^1^H NMR was as previously described (Sun et al., 2020b). Briefly, spectra were acquired on Bruker AV 600 MHz superconducting Fourier transform NMR spectrometer (Bruker, Germany). On MestReNova 6.1.1 software (Mestrelab Research, USA), TSP (δ0 ppm) was used as a reference, in the 0–10 ppm range, with a width of 0.004 ppm, eliminating the water peak (4.6–5.2 ppm). After normalization, PCA and OPLS-DA were performed on SIMCA-P13.0 (UMERICS, UMEA, Sweden). At the same time, 7-round cross validation and permutation tests (200 permutations) were used to verify whether the model was over-fitted. Metabolites with variable importance plot (VIP) > 1.0 at multivariate statistical analysis and p < 0.05 at univariate statistics were identified as the final differential metabolites. Combine online databases HMDB (http://www.hmdb.ca/) and BMRB (https://bmrb.io/metabolomics/) to identify biomarkers. At last, the markers were imported into MetaboAnalyst 5.0 (https://www.metaboanalyst.ca/) for metabolic pathway analysis.

### 2.11 Measurement of SCFAs in cecum

The content of SCFAs (acetic acid, propionic acid, isobutyric acid, butyric acid, isovaleric acid, valeric acid, caproic acid) was determined by GC-MS. Detailed experimental procedures were provided in Supplementary Materials.

### 2.12 Statistical analysis

All data were expressed as mean ± standard deviation. SPSS (version 19.0) was used to analyze the normal distribution and variance homogeneity of data. The differences between the two groups were compared by one-way ANOVA. Through welch’s *t*-test, the species with significant differences among groups in 16S rRNA analysis and metabolic pathways with significant differences in function prediction were determined. *p* < 0.05 was considered to be statistically significant. Correlations between gut microbiota and other experimental parameters were determined by spearman’s correlation test and visualized by R (version 3.3.1).

## 3 Results

### 3.1 Effects of SGD on body weight and organ index of CCl_4_-treated rats

As shown in Supplementary Figure S1A, the body weight of the rats in each group increased with time. However, the weight gain trend of the liver-injured rats was significantly smaller than that of the normal rats throughout the experiment. From the second week, the body weight of liver-injured rats was significantly different from that of normal rats. While LIM, SGDT, PLIM and PSGDT showed a similar growth trend in rat body weight. The organ index results showed that the liver index and spleen index were significantly increased in LIM compared with Con, while SGD treatment could significantly reduce the liver index. Similar results were seen in PLIM and PSGDT given antibiotics. Compared with normal rats, the liver index and spleen index of PLIM were significantly increased, but not different from LIM, while the liver index of PSGDT was significantly decreased (Supplementary Figure S1B,C). However, none of the thymus indices changed significantly compared with Con (Supplementary Figure S1D). This result showed that CCl_4_-induced liver injury seriously affected the weight gain of rats and the normal weight ratio of liver and spleen with or without antibiotics. SGD treatment has therapeutic effect on liver.

### 3.2 SGD ameliorates CCl_4_-induced liver injury and correlates with gut microbiota

The results of H&E staining were shown in Supplementary Figure S2A. The hepatocytes in Con were morphologically intact without degeneration and damage. However, in LIM, massive fibrous connective tissue hyperplasia, inflammatory cell infiltration and mild vacuolar degeneration were seen in the liver tissue. Compared with LIM, the degree of fibrosis of hepatocytes in SGDT was reduced, and the vacuolar degeneration and inflammatory cell infiltration decreased significantly. PLIM and PSGDT showed similar results to LIM and SGDT. Briefly, lesions were found in PLIM, which were alleviated in PSGDT. Interestingly, PLIM had more severe damage than LIM, while PSGDT did not recover as much as SGDT.

The results of ALT, AST, ALB and TP were shown in Supplementary Figure S2B. At the second week, compared with Con, ALT and AST of LIM, SGDT, PLIM and PSGDT increased significantly. This implies the successful establishment of liver injury model. At the eighth week, compared with LIM, ALT and AST in SGDT group decreased significantly. However, there was no significant difference in ALT and AST between PSGDT and PLIM. During the whole experiment, there was no significant difference in ALB and TP among the groups.

The antioxidant effects of SGD in the liver were shown in Supplementary Figure S2C. Compared with Con, liver MDA and CYP2E1 levels were significantly increased, while SOD and GSH levels were significantly decreased in LIM. This indicated that CCl_4_ had activated CYP2E1 in rats to overexpress, thereby causing oxidative stress in the liver. SGD treatment significantly reduced MDA and CYP2E1, and restored SOD and GSH levels, significantly attenuating oxidative stress in liver-injured rats. In addition, these of PLIM were similar to LIM, while there were no differences with PLIM in PSGDT.

The results suggested that gut microbiota might be protective against chemical-induced liver injury. SGD could reduce the severity of CCl_4_-induced liver injury through antioxidant effects, which might be related to its regulation of gut microbiota.

### 3.3 Regulation of SGD on inflammatory cytokines in rats with CCl_4_-induced liver injury

As can be seen from the measurement of plasma inflammatory cytokines (Supplementary Figure S3), the levels of IL-6 and TNF-α were significantly increased in LIM compared with Con, and decreased after SGD treatment. This indicated that CCl_4_-induced liver injury caused inflammatory responses in rats, while SGD had anti-inflammatory effects. Strangely, inflammatory cytokines were not elevated in PLIM, a liver injury model group given antibiotics. Likewise, there was no increase in PSGDT. It may be that the antibiotics eliminated a large number of harmful microorganisms, thereby reducing the inflammatory response in the rats.

### 3.4 16S rRNA gene sequencing analysis of fecal samples

#### 3.4.1 Regulation of SGD on the structure of gut microbiota in CCl_4_-induced liver injury

28 fecal samples (4 in the Con group and six in each of the remaining groups) yielded 1,321,066 valid sequences with 47,181 reads per sample. And the number of effective bases was 548,333,708, the average length of the sequence was 415. With 97% similarity, 1,061 OTUs were detected. From the rarefaction curves, it could be seen that the curves all tended to be smooth with the increase in the number of sample reads (Supplementary Figure S4A,B), indicating that the sequencing depth in this experiment was reasonable. In addition, it could be seen from the rank-abundance curve that with the increase of OTU ranking, the curve width and smoothness of LIM were smaller than those of Con, while SGDT was closer to Con (Supplementary Figure S4C). The results of alpha diversity were shown in Supplementary Table S2 and Supplementary Figure S4D–H. The coverage index of each group was greater than 0.99, which means the sampling was satisfactory. What’s more, there was no significant difference in Chao index and Simpson index between Con and LIM, while the Shannoneven index and Shannon index of LIM were significantly lower than those of Con. Compared with LIM, the Shannoneven index and Shannon index were significantly increased in SGDT. These results indicated that CCl_4_ treatment could cause a decrease in the diversity and evenness of the gut microbial community in rats, while SGD treatment could significantly improve the diversity in liver-injury rats.

Then, beta diversity analysis was performed. PCA analysis showed that the microbial communities of the Con, LIM and SGDT had obvious separation. From the distribution of the different groups on PC1 axis, SGDT tended to be closer to Con than LIM (Supplementary Figure S5A,B). Furthermore, the results of PCoA and NMDS were similar to those described above, with partial overlap between SGDT and Con (Supplementary Figure S5C–E). All of these had indicated that CCl_4_-induced liver injury caused changes in the structure of gut microbiome, which were ameliorated by SGD treatment. The stress value in NMDS was 0.08, less than 0.1, which indicated that it could accurately reflect the true distribution of data ordering. Heatmap and hierarchical clustering showed that each group could be basically classified into one category and had differences (Supplementary Figure S5G,H). The weighted_unifrac-based ANOSIM (R = 0.3492, *p* = 0.003, Supplementary Figure S5F) and the bray-curtis-based PERMANOVA (*R* ^2^ = 0.37114, *p* = 0.001) showed that (the number of permutations for both was 999) the intra-group difference was lower than the inter-group difference.

The differences of gut microbiome composition among different groups were analyzed at the level of phylum, class, order, family and genus. The results were shown in Supplementary Figure S6–10. At each classification level, major components were presented in the bar and pie charts and differences were visualized via heatmaps. Welch’s *t*-test showed that the composition of the microbiota was significantly changed in LIM compared with Con, which was recovered after SGD treatment. The result demonstrated that SGD had the function of regulating gut microbiota.

**FIGURE 1:**
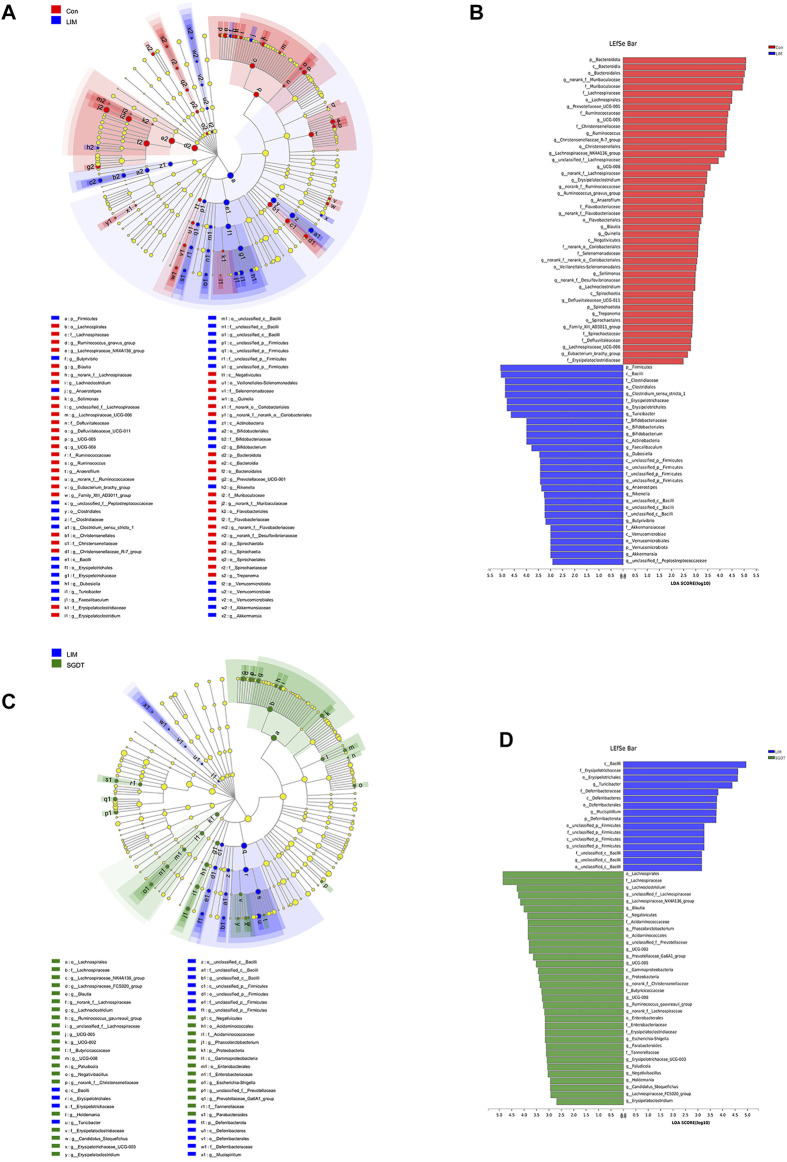
Key bacterial taxa significantly altered in CCl_4_-induced liver injury and SGD treatment. **(A)** Cladogram generated by LEfSe analysis between Con and LIM. **(B)** LDA scores of enriched taxa in LEfSe analysis between Con and LIM. **(C)** Cladogram generated by LEfSe analysis between LIM and SGDT. **(D)** LDA scores of enriched taxa in LEfSe analysis between LIM and SGDT.

More critically, we also performed LEfSe analysis to identify bacterial taxa that most likely accounted for differences between groups at the genus level (Shi et al., 2020). The taxa with LDA≥2 were considered to be significantly different. Across all taxonomic levels, 76 bacterial taxa with significant differences were identified between Con and LIM, including 37 at the genus level, and 50 between LIM and SGDT, including 26 at the genus level (Figure 1). After exclusion of duplicate taxa, 52 clusters were considered to be marker bacterial taxa with significant changes during establishment of the liver injury model and treatment with SGD. Compared with Con, 12 genera in LIM were significantly increased, while 25 genera were decreased. After treatment, 11 genera recovered to varying degrees. Specifically, treatment increased the abundance of Blautia, Erysipelatoclostridium, UCG-005, UCG-008, Lachnoclostridium, Lachnospiraceae_NK4A136_group, norank_f__Lachnospiraceae and unclassified_f__Lachnospiraceae, while decreased Turicibacter, g__unclassified_c__Bacilli and g__unclassified_p__Firmicutes. In addition, 15 bacterial genera showed significant increases or decreases after dosing. The results were presented in Supplementary Table S3. The results were corroborated with welch’s *t*-test, which further indicated that liver injury caused the gut microbiota disturbance in rats, and SGD had a regulatory effect on the gut microbiota of rats.

#### 3.4.2 Correlation network analysis

There were extensive interactions among gut microbiota. In order to explore the changes of gut microbiota correlations in liver injury and the effect of SGD on them, correlation networks were constructed by spearman for the top 50 genera in each group. The correlation network graph for each group was shown in Supplementary Figure S11 to demonstrate the relatedness of genus, and the degree of correlation was also shown by heatmap (Supplementary Figure S12). Transitivity (Con: one; LIM: 0.476; SGDT: 0.629) could measure the degree of association of each node in the network. The results showed that Con had the highest transmissibility, while LIM was the lowest, and SGDT was between the two, indicating that gut microbiota in normal rats had a high correlation, which was reduced by CCl_4_, and SGD treatment could restore this correlation to a certain extent. It could be known from the network that Alloprevotella, Blautia, Prevotellaceae_UCG-001, UCG-003 and unclassified_f__Prevotellaceae in Con had significant positive correlations with each other, while they all had negative correlations with Ruminococcus_torques_group. Additionally, Romboutsia and UCG-005 were positively correlated. However, these correlations were disrupted in LIM, where Alloprevotella and Prevotellaceae_UCG-001 were found to be significantly negatively correlated with Turicibacter. Romboutsia had high topological properties (DC = 0.152, CC = 0.283, BC = 0.196) in LIM, which played a central role in the network, however, it was significantly negatively correlated with Lachnospiraceae_NK4A136_group. It was speculated that the decrease of Lachnospiraceae_NK4A136_group in LIM might be related to it. Fortunately, these interactions were recovered in SGDT, in which Alloprevotella has high topological properties (DC = 0.208, CC = 0.231, BC = 0.052), so it is considered to be the predominant microbiota in the recovery correlation after SGD treatment. Furthermore, SGD strengthened the positive correlation of Alloprevotella with Parabacteroides, Prevotellaceae_Ga6A1_group and Ruminococcus_gauvreauii_group.

#### 3.4.3 PICRUSt functional prediction

**FIGURE 2:**
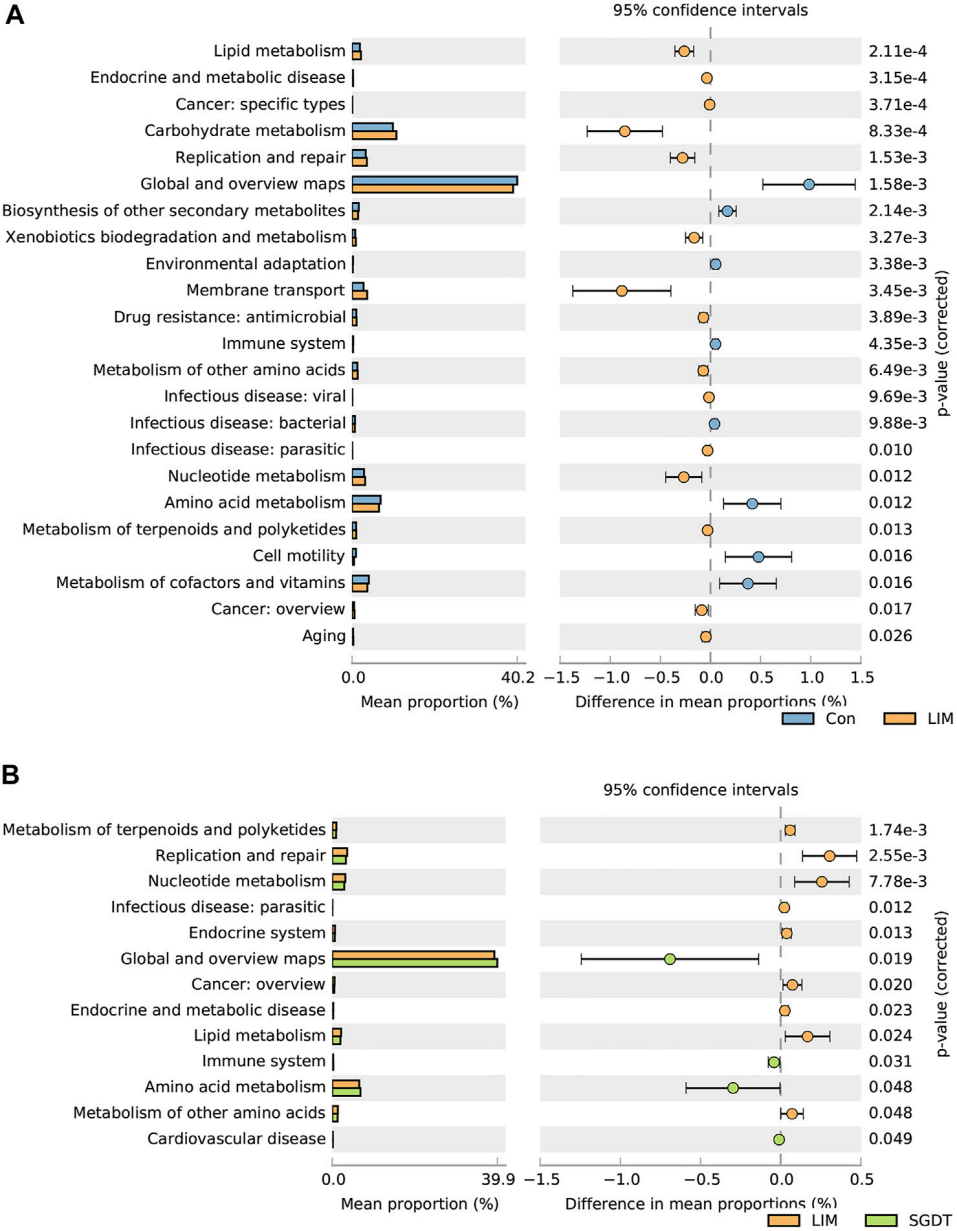
Comparison of gut microbiota functional pathways between Con and LIM **(A)**, and between LIM and SGDT **(B)**.

In order to explore the changes in gut microbiota function after treatment with CCl_4_ and SGD, PICRUSt predictions were performed on 16S rRNA data. Compared with Con, 23 pathways were significantly different in LIM. In the metabolic pathway, genes for amino acid metabolism, biosynthesis of secondary metabolites, and vitamin metabolism were decreased, while genes for glucose metabolism, lipid metabolism, terpenoid metabolism, nucleotide metabolism, and biodegradation of exogenous chemicals were more abundant. Suggesting that these metabolic pathways were disrupted in liver injury. Notably, 13 pathways were significantly different after administration, in which terpenoid metabolism, nucleotide metabolism, lipid metabolism, and amino acid metabolism were all restored (Figure 2).

#### 3.4.4 Establishment of PGF model

The alpha diversity indices (Chao index and Shannon index) were significantly decreased in the PLIM group and PSGDT group (Supplementary Table S2). This indicated that antibiotic treatment could significantly reduce the community richness and diversity of gut microbiota in rats. Moreover, from the Veen plot, the number of OTUs for PLIM and PSGDT was significantly lower than the other three groups. PCoA and NMDS analysis showed that PLIM and PSGDT were significantly different from the LIM. In addition, the abundance and proportion of gut microbiota were decreased in both the PLIM and PSGDT at the phylum, class, order, family, and genus levels (Supplementary Figure S13). These results showed that a large number of bacteria in the gut of rats could be cleared after antibiotic treatment, indicating that PGF model had been successfully established.

### 3.5 Regulation of fecal metabolites by SGD in rats with liver injury

**FIGURE 3:**
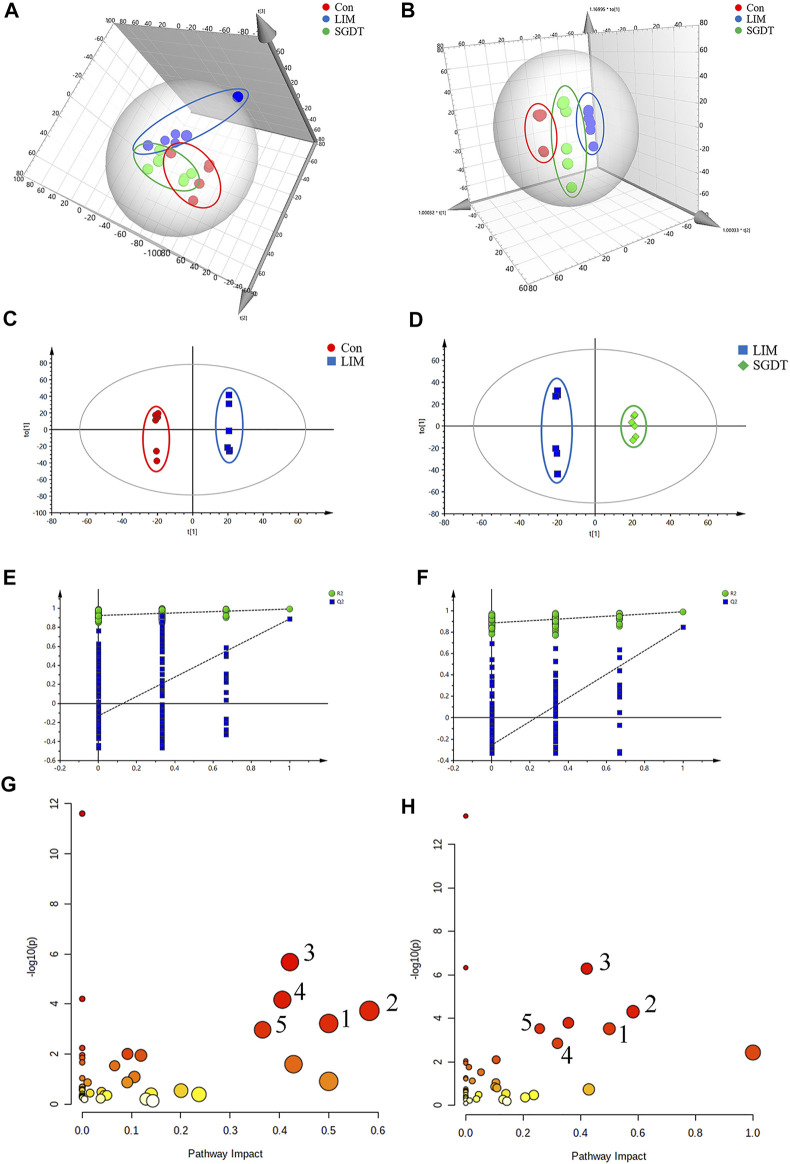
Fecal metabolite profiling based on ^1^H NMR spectrum of fecal samples, and metabolic pathways. **(A,B)** PCA and OPLS-DA score plots of Con, LIM and SGDT. **(C,D)** OPLS-DA score plots between Con and LIM, LIM and SGDT. **(E,F)** Permutation test plots (200 permutations) between Con and LIM (*R* ^2^ = 0.925, Q^2^ = -0.13), LIM and SGDT (*R* ^2^ = 0.89, Q^2^ = -0.227). **(G,H)** Significant metabolic pathways between Con and LIM, LIM and SGDT. (1) d-Glutamine and D-glutamate metabolism, (2) Alanine, aspartate and glutamate metabolism, (3) Arginine biosynthesis, (4) *Glycine*, serine and threonine metabolism, (5)Arginine and proline metabolism.

Metabolomics analysis of fecal samples was performed by ^1^H NMR (Supplementary Figure S14). 78 endogenous metabolites were detected (Supplementary Table S4). From the score plots of PCA and OPLS-DA (Figures 3A,B), it can be seen that there was a clear distinction between Con and LIM, and SGDT was more closely related to Con than LIM. This indicated that the fecal metabolic pattern of CCl_4_-induced liver injury was significantly altered, whereas SGDT was more similar to Con than that of LIM, suggesting that SGD had a therapeutic effect on liver injury. To further analyze the differential metabolites, OPLS-DA analysis was performed between Con and LIM, and between LIM and SGDT. Similar results were obtained, with significant changes in metabolites for LIM versus Con and SGDT versus LIM (Figures 3C,D). 7-rounds of cross validation (Supplementary Table S5) and permutation tests (Figures 3E,F) demonstrated that the models were stable and accurate in prediction.

Based on the loading plots from OPLS-DA analysis (Supplementary Figures S15A,B), and VIP >1, *p* < 0.05, 51 significantly changed differential metabolites were identified between Con and LIM. In addition, 41 differential metabolites were identified in LIM and SGDT. Among them, 37 differential metabolites were reversed by SGD treatment after significant changes in liver injury (Supplementary Table S6). Therefore, these 37 differential metabolites were considered to be key fecal metabolites for SGD in regulating CCl_4_-induced liver injury.

**FIGURE 4:**
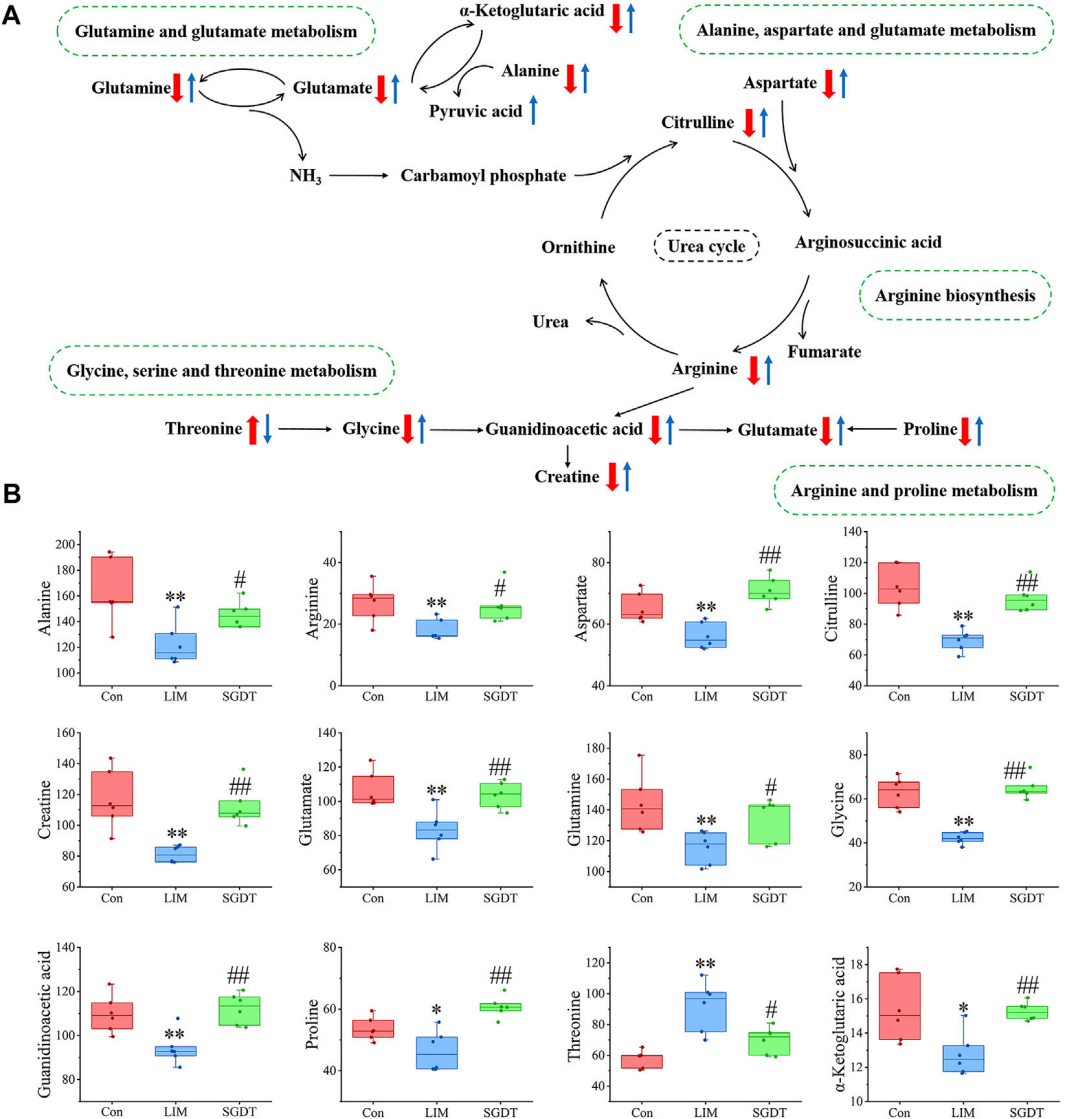
Schematic diagram of key metabolic pathways **(A)** and level changes of corresponding metabolites **(B)**. The "↑" or "↓" in **(A)** represents the differential metabolite which was significantly up-regulated or down-regulated, with red as compared with Con and blue as compared with LIM. In **(B)**, *p < 0.05, **p < 0.01, compare with Con; #*p* < 0.05, ##*p* < 0.01, compare with LIM.

The most meaningful metabolic pathways between Con and LIM, and between LIM and SGDT were analyzed using MetaboAnalyst 5.0. As shown in Figures 3G,H, these differential metabolites were associated with multiple amino acid metabolic pathways, which was consistent with the results of PICRUSt analysis. Among them, D-glutamate and D-glutamate metabolism, Alanine, Aspartate and glutamate metabolism, Arginine biosynthesis, *Glycine*, Serine and Threonine metabolism, Arginine and proline metabolism were the most relevant (*p* < 0.05, impact value >0.10), suggesting that these five metabolic pathways were the key pathways of SGD in the treatment of liver injury. The relationships between key metabolic pathways and the changes in corresponding metabolites were shown in Figure 4.

In addition, the role of gut microbiota in the effect of SGD on fecal metabolites was investigated by analyzing PGF rats. From the score plots of PCA and OPLS-DA (Supplementary Figures S15C,D), both PLIM and PSGDT are clearly distinguishable from LIM. Among them, PLIM and PSGDT were not clearly separated in PCA. This indicated that the absence of gut microbiota caused significant changes in fecal metabolites, while the difference between PLIM and PSGDT, which were also PGF models, may not be very significant.

Differential metabolites between LIM and PLIM and between PLIM and PSGDT were analyzed by OPLS-DA. The results were shown in Supplementary Figure S15E,F, and the validation results of the model were shown in Supplementary Table S5 and Supplementary Figure S15G,H. There were significant differences between LIM and PLIM, suggesting that the reduction in gut microbiota further caused significant changes in fecal metabolites. According to the loading plots analyzed by OPLS-DA (Supplementary Figure S15I,J), VIP >1 and *p* < 0.05, 12 significantly changed differential metabolites were identified between LIM and PLIM. And nine differential metabolites were found in PLIM and PSGDT, of which one was SGD that reversed the metabolites of the PLIM group, and three were that reversed the differential metabolites between Con and LIM (Supplementary Table S7). This suggests that in PGF, SGD can only reverse four disordered metabolites, one caused by loss of gut microbiota and three caused by CCl_4_-induced liver injury. Compared with the 37 differential metabolites recovered in SGDT, this further confirmed that SGD-recovered fecal metabolites were associated with gut microbiota.

### 3.6 Effects of SGD treatment on SCFAs in cecum contents of rats with liver injury

**FIGURE 5:**
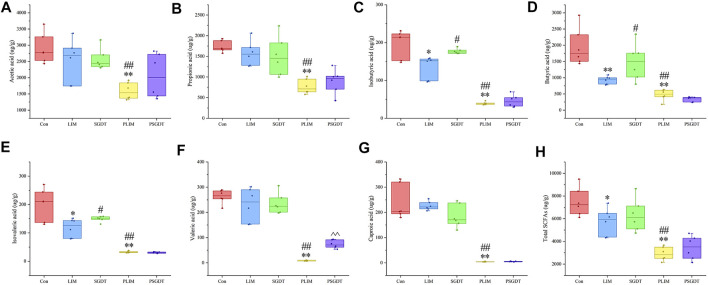
Contents of SCFAs in cecum contents of rats in each group. **(A)** Acetic acid **(B)** Propionic acid **(C)** Isobutyric acid **(D)** Butyric acid **(E)** Isovaleric acid **(F)** Valeric acid **(G)** Caproic acid **(H)** Total SCFAs. *p < 0.05, **p < 0.01, compare with Con; #*p* < 0.05, ##*p* < 0.01, compare with LIM; ^ ^ *p* < 0.01, compare with PLIM.

Due to strong volatility, the content of SCFAs in cecum of rats was determined by GC-MS. Typical TICs were shown in Supplementary Figure S16. As shown in Figure 5, compared with Con, the total SCFAs in LIM were significantly reduced, in which the contents of isobutyric acid, butyric acid and isovaleric acid were significantly decreased in LIM, while acetic acid, propionic acid, valeric acid and caproic acid were not significantly different. SGD treatment significantly restored isobutyric acid, butyric acid, and isovaleric acid levels. The results showed that CCl_4_-induced liver injury led to disturbance of SCFAs in the rat gut, which was significantly ameliorated by SGD. Moreover, all measured SCFAs were significantly reduced in PLIM compared to Con and LIM. Whereas isobutyric acid, butyric acid, and isovaleric acid (recovered in SGDT) were not significantly different in PSGDT and PLIM, and in PGDT group, except for the addition of valeric acid, the others were not different from PLIM. This indirectly proves that the gut microbiota was significantly correlated with SCFAs, and the gut microbiota plays an important role in the recovery of SCFAs by SGD. Furthermore, PCA and OPLS-DA analysis showed that Con was significantly separated from LIM, while SGDT had a tendency to be close to Con, however, PLIM and PSGDT were more clearly separated from Con (Supplementary Figure S17). This proves once again that the gut SCFAs of rats with liver injury were disturbed, and the presence of gut microbiota was required for the therapeutic effect of SGD. The methodological validation results were shown in Supplementary Figure S16 and Supplementary Table S8–10, which indicate that the assay was reliable.

### 3.7 Correlation analysis of gut microbiota with fecal metabolites and SCFAs

#### 3.7.1 Correlation of gut microbiota and fecal metabolites

**FIGURE 6:**
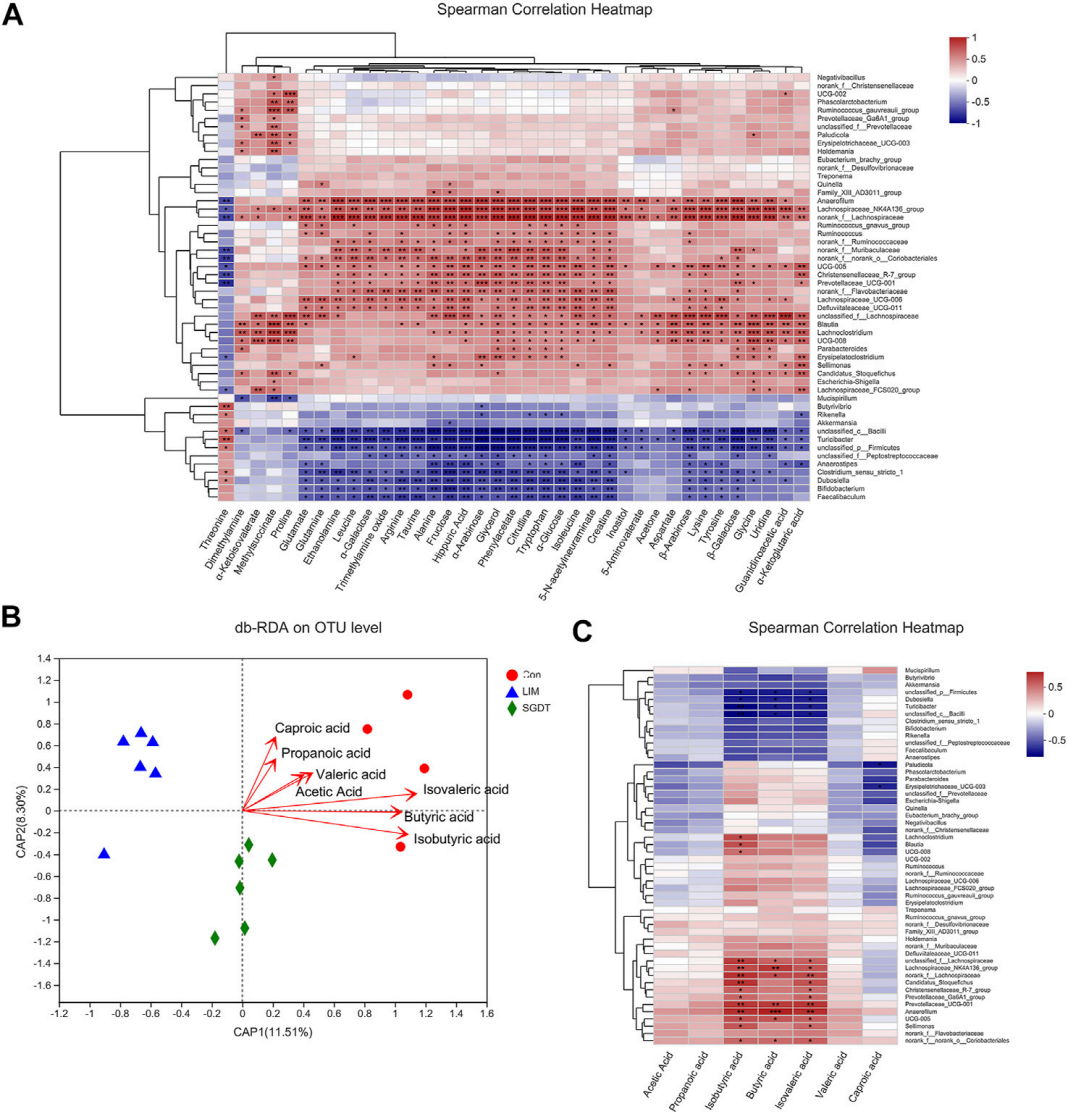
Correlation analysis of gut microbiota with SCFAs and fecal metabolites. **(A)** Spearman’s correlation analysis of 52 marker bacterial taxa and 37 fecal metabolites. **(B)** db-RDA analysis of gut microbiota and SCFAs. **(C)** Spearman’s correlation analysis of 52 marker bacterial taxa and seven SCFAs. *p < 0.05, **p < 0.01, ***p < 0.001.

Spearman’s correlation analysis was performed on 37 fecal metabolites that could be reversed by SGD and 52 marker bacterial taxa. The results showed broad correlations between these fecal metabolites and marker bacterial taxa (Figure 6A). The results showed broad correlations between these fecal metabolites and marker bacterial taxa. Glutamate, glutamine, alanine and α-ketoglutarate, associated with key metabolic pathways, were all positively correlated with Lachnospiraceae_NK4A136_group, norank_f__Lachnospiraceae, UCG-005 and unclassified_f__Lachnospiraceae, while negatively with Anaerostipes, Turicibacter, unclassified_c__Bacilli and unclassified_p__Firmicutes. Citrulline, aspartic acid, arginine were all positively with Anaerofilum, Blautia, Lachnospiraceae_NK4A136_group, Lachnospiraceae_UCG-006, norank_f__Lachnospiraceae and UCG-005, but negatively with Turicibacter and unclassified_c__Bacilli. *Glycine* and proline were positively with Blautia, and Erysipelatoclostridium was positively with glycine but negatively with threonine. Guanidinoacetic acid and creatine were positively with Lachnoclostridium.

#### 3.7.2 Correlation between gut microbiota and SCFAs

Based on the data of gut microbiota and SCFAs, db-RDA (unweighted_unifrac) was used to analyze the correlation between gut microbiota and SCFAs in SGD-treated liver-injured rats. As shown in Figure 6 B, acute angles indicated a positive correlation between SCFAs, of which Butyric acid, Isobutyric acid and Isovaleric acid were the most relevant SCFAs for liver injury, followed by acetic acid, Propanoic acid, Valeric acid and Caproic acid. Correlations between the identified 52 marker bacterial taxa and SCFAs were analyzed by spearman. The results showed that 20 marker taxa were closely related to SCFAs, of which 14 showed significant positive correlation, namely Lachnoclostridium, Blautia, UCG-008, unclassified_f__Lachnospiraceae, Lachnospiraceae_NK4A136_group, norank_f__Lachnospiraceae, Candidatus_Stoquefichus, Christensenellaceae_R-7_group, Prevotellaceae_Ga6A1_group, Prevotellaceae_UCG-001, Anaerofilum, UCG-005, Sellimonas and norank_f__norank_o__Coriobacteriales. In contrast, six were significantly negatively correlated, namely unclassified_p__Firmicutes, Dubosiella, Turicibacter, unclassified_c__Bacilli, Paludicola and Erysipelotrichaceae_UCG-003 (Figure 11 6). The SCFAs closely related to the gut microbiota were isobutyric acid, butyric acid and isovaleric acid, which was consistent with the db-RDA analysis.

## 4 Discussion

Numerous studies support the causal role of gut microbiota in the development and progression of liver disease. In this study, we investigated the therapeutic effect of SGD, while also exploring the role of the gut microbiota in the development of liver injury and the efficacy of SGD through PGF rats. More importantly, we assessed the regulatory effect of SGD on CCl_4_-induced imbalances in gut microbiota, fecal metabolites, and SCFAs.

### 4.1 Therapeutic effect of SGD on CCl_4_-induced liver injury

Studies have shown that the intake of CCl_4_ can overexpress CYP2E1, which in turn converts CCl_4_ into toxic free radicals (Zhang et al., 2018; Yao et al., 2020). This experiment has consistent results, the level of hepatic CYP2E1 was increased in LIM, and histological showed that the liver cells of LIM were massively proliferated and degenerated. However, after treatment, the level of CYP2E1 decreased, the lesions of hepatocytes were alleviated, and the liver index reduced. At the same time, when liver cells are damaged, the important metabolic enzymes ALT and AST are released into the blood (Kwo et al., 2017). After treatment, the decreased levels of ALT and AST in serum confirmed that SGD protects against CCl_4_-induced liver injury. MDA is the end product of the peroxidation reaction (Tsikas, 2017). SOD and GSH are important antioxidant enzymes against reactive oxygen species (Liu et al., 2020). In this experiment, CCl_4_ caused the increase of MDA *in vivo* and consumed a large amount of SOD and GSH, which was consistent with the previous report (Zhang et al., 2021b), while SGD treatment prevented the increase of MDA and restored the levels of SOD and GSH. This proved that SGD could protect CCl_4_-induced liver injury against oxidation. Notably, from the above indicators, the PGF model group (PLIM) that was given antibiotics to eliminate a large number of gut microbes exhibited more severe liver injury, while there was no significantly recovered in the corresponding treatment group (PSGDT). Therefore, we speculate that gut microbiota has a protective effect on CCl_4_-induced liver injury, and the efficacy of SGD may be related to its regulation of gut microbiota.

Dysregulation of the gut microbiota and its metabolites increases gut permeability, which interacts with the host immune system to cause inflammation (Leung et al., 2016; Tilg et al., 2016; Jones and Neish, 2021). After hepatocyte injury, some gut-derived factors can activate Kuffer cells to secrete TNF-α, and IL-6 (He and Karin, 2011; Schmidt-Arras and Rose-John, 2016; Kim et al., 2019). In this study, the levels of IL-6 and TNF-α in LIM increased. This may be due to liver injury allowing bacterial metabolites to enter the rats. The decrease after treatment suggests that SGD has an anti-inflammatory effect. However, IL-6 and TNF-α in both PLIM and PSGDT remained unchanged compared to Con. This may be due to the elimination of a large number of microorganisms by antibiotics, which leads to a reduction in the production of bacterial metabolites. Harmful bacterial products entering the body were reduced, thereby reducing the inflammatory response.

Collectively, in the presence of gut microbiota, SGD can treat CCl_4_-induced liver injury by inhibiting the over-expression of CYP2E1 to reduce oxidative stress, enhancing antioxidant enzymes to eliminate free radicals, and reducing inflammation.

### 4.2 Effects of SGD on the composition and correlation network of gut microbiota in CCl_4_-induced liver injury

To explore whether SGD could modulate the gut microbiota, we performed 16S rRNA sequencing. ANOSIM and PERMANOVA analyses demonstrated that grouping was meaningful. The results revealed distinct differences in the composition of gut microbiota in Con, LIM, and SGDT, while SGDT was more similar to Con than to LIM. The group difference analysis based on welch’s *t*-test showed that the composition of LIM and Con was different, while SGD had a therapeutic effect. 52 marker bacterial taxa at the genus level were identified by further LEfSe analysis. In contrast, 11 of the 37 bacterial taxa significantly altered in LIM were reversed by SGD. Studies have shown that Blautia can degrade dietary components that are not digested by the host and increase the production of SCFAs, thereby promoting food digestion and improving energy intake (Liu et al., 2021). As a dominant genus, Blautia is closely related to the health of the host and can maintain intestinal environmental balance and prevent inflammation by upregulating intestinal regulatory T cells (Kim et al., 2014). Lachnoclostridium is a producer of butyric acid, which inhibits the proliferation of pathogens and relieves intestinal inflammation (Shang et al., 2020). In addition, the same butyrate-producing Lachnospiraceae_NK4A136_group can maintain the integrity of the intestinal barrier (Ma et al., 2020). The reduction of these beneficial bacteria in LIM is undoubtedly one of the reasons for worsening liver injury. However, Turicibacter was significantly elevated in LIM. According to reports, Turicibacter is involved in fermentative metabolism to produce lactic acid, which is positively correlated with serotonin in the gut, and serotonin is involved in liver regeneration (Kamimura et al., 2018; Fung et al., 2019). It is speculated that serotonin levels are elevated during regeneration following liver injury in LIM, resulting in elevated Turicibacter levels. Notably, treatment with SGD altered 15 other bacteria in addition to reversing the microbiota described above (Supplementary Table S3). Studies have shown that Mucispirillum has a positive correlation with intestinal inflammation. Its level in SGDT was reduced, again demonstrating the efficacy of SGD in reducing intestinal inflammation (Herp et al., 2021). Parabacteroides have been demonstrated to regulate metabolic dysfunction by increasing succinic acid and secondary bile acids, and have the ability to regulate inflammatory markers to promote intestinal barrier integrity (Wang et al., 2019; Koh et al., 2020). It is increased in SGDT, suggesting that SGD has a role in regulating bile acids and intestinal barriers. SGD can also increase the abundance of Phascolarctobacterium, which has been reported to be beneficial to limit the growth of the pathogenic bacteria Clostridioidies *difficile* to alleviate intestinal inflammation (Nagao-Kitamoto et al., 2020).

Correlation network analysis showed that CCl_4_ led to a weakened interaction of the rat gut microbiota. Positive interactions were found in Alloprevotella, Blautia and Prevotellaceae_UCG-001 in normal rats, while they were negatively correlated with Ruminococcus_torques_group. In addition to Blautia that can produce SCFAs, there are also Alloprevotella and Prevotellaceae_UCG-001, so it is speculated that their positive correlation is one of the ways to promote the production of SCFAs by the gut microbiota (Kong, Gao, Yan, Huang, Qin; Xiao et al., 2020). There is evidence that Ruminococcus_torques_group is positively associated with inflammation (Zheng et al., 2020). Their negative correlation may be related to the maintenance of the stability of the gut microbiota by suppressing inflammation. However, their relationship was broken in LIM. More importantly, both Alloprevotella and Prevotellaceae_UCG-001 were found to be negatively correlated with Turicibacter. And Turicibacter has been detected to be elevated in LIM. Therefore, it was speculated that Turicibacter inhibited the production of SCFAs by Alloprevotella and Prevotellaceae_UCG-001. After treatment, microbiota interactions were restored. SGD recovered the association of Alloprevotella with others and strengthened its positive association with Parabacteroides, Prevotellaceae_Ga6A1_group and Ruminococcus_gauvreauii_group. It was detected that SGD increased the levels of these three bacteria, among which Prevotellaceae_Ga6A1_group was detected to have a significant positive correlation with SCFAs, and its positive correlation with Alloprevotella may be one of the factors for the recovery of SCFAs by SGD. In addition, studies have shown that Romboutsia plays a key role in maintaining host health, and UCG-005 interacts with it to promote the production of SCFAs (Mangifesta et al., 2018). Both correlations and levels of UCG-005 were decreased in LIM. In SGDT, UCG-005 was significantly positively correlated with Christensenellaceae_R-7_group. Both of these were detected to be significantly positively correlated with SCFAs, and levels of UCG-005 recovered after treatment. These results all suggest that SGD has a role in regulating gut microbiota.

PICRUSt analysis showed that SGD treatment could restore disordered terpenoid metabolism, nucleotide metabolism, lipid metabolism and amino acid metabolism. Liver disease leads to disturbances in bile acid metabolism, which may allow more lipids to accumulate in the gut, leading to elevated lipid metabolism (Li and Chiang, 2014; Guo et al., 2018). The bacterial metabolites of tryptophan (indole and indole -3- propionic acid) have been shown to reduce liver injury by increasing gut integrity and reducing pro-inflammatory cytokines (Schoeler and Caesar, 2019; Zhao et al., 2019; Chen and Vitetta, 2020). The results showed that intestinal flora metabolism was closely related to liver injury, and SGD had a restoring effect on abnormal metabolic pathways.

In addition, the successfully established PGF model confirmed that CCl_4_ induced more severe liver injury in the absence of gut microbiota, and the therapeutic effect of SGD was attenuated. The results also proved that gut microbiota had a protective role in the formation of CCl_4_-induced liver injury, and SGD could treat liver injury by regulating gut microbiota.

### 4.3 Effects of SGD on fecal metabolites and their correlation with gut microbiota in CCl_4_-induced liver injury

Intestinal dysbiosis affects host metabolism by altering metabolites, thereby contributing to the development of liver disease. PICRUSt has predicted changes in metabolic pathways. For further analysis of specifically altered metabolites, fecal metabolomics analysis was performed. From the metabolic pathway analysis, it was known that the differential metabolites were associated with a variety of amino acid metabolic pathways. SGD significantly corrected five of them (Figure 4). To explore the effect of gut microbiota on liver injury by altering host metabolism and the therapeutic mechanism of SGD, spearman analysis was performed (Figure 6A).

Glutamine can inhibit the activation of inflammatory cytokines (Kim and Kim, 2017; McCarty and Lerner, 2021). In this study, the levels of glutamine, glutamate, alanine, and α-ketoglutarate were decreased in LIM, indicating abnormal transport of ammonia in the gut. This will increase the production of ammonia and the amount of it entering the body through the portal vein. Hyperammonemia is an important cause of acute liver failure (Gupta et al., 2016). In addition, LIM has severe inflammation and oxidative stress, which may be related to it, and recovered after treatment.

The urea cycle involving citrulline, aspartic acid, and arginine converts ammonia into urea. Arginine can increase cell migration through NO to rapidly restore mucosal damage (Rhoads et al., 2004). They drop in LIM, which may interfere with the urea cycle, leading to the accumulation of ammonia. Moreover, it may also affect the repair of intestinal mucosal damage. All of the above metabolites were elevated after treatment, suggesting that SGD is beneficial for improving ammonia transport and the urea cycle, thereby reducing the accumulation of ammonia.

The deficiency or excess of threonine can reduce the synthesis of intestinal mucosal proteins and mucins, thereby affecting the intestinal mucosal barrier (Wang et al., 2007). Meanwhile, glycine attenuates apoptosis and proline protects the barrier from oxidative damage (Wolfarth et al., 2020). In this study, their levels were decreased in LIM, which may affect the expression of intestinal barrier-related proteins. However, there was some recovery after treatment, suggesting that SGD may treat liver injury by improving the intestinal barrier.

Liver disease causes changes in energy metabolism (Traussnigg et al., 2017). Guanidinoacetic acid is a precursor of creatine. Creatine can quickly provide energy for the body and play an important role in maintaining intestinal homeostasis. Decreased levels of guanidinoacetic acid and creatine indicate disturbances in the body’s energy metabolism, and SGD can modulate them.

Correlation analysis indicated that metabolites related to ammonia transport and urea cycle were positively correlated with Lachnospiraceae_NK4A136_group and negatively correlated with Turicibacter. Studies have shown that a diet supplemented with α-ketoglutarate increases the Lachnospiraceae_NK4A136_group in the gut and reduces the concentration of ammonia in the gut (Chen et al., 2018). Ammonia had a significant positive correlation with Turicibacter (Khan et al., 2021). In this study, Lachnospiraceae_NK4A136_group decreased and Turicibacter increased, which may be related to decreased ammonia transport and urea cycle-related metabolites. This was reversed after treatment, suggesting that SGD could promote ammonia excretion by improving ammonia transport and the urea cycle.

In studies on intestinal diseases, Blautia and Erysipelatoclostridium were found to be significantly associated with the intestinal mucosa (Richard et al., 2018; Nagayama et al., 2020). In this study, glycine and proline in LIM were positively correlated with Blautia, Erysipelatoclostridium was positively correlated with glycine and negatively correlated with threonine. The levels of Blautia, Erysipelatoclostridium, and glycine, proline, threonine were restored after treatment. It is speculated that SGD can affect the synthesis of glycine, proline and threonine-related proteins by acting on Blautia and Erysipelatoclostridium, thereby restoring the integrity of the intestinal mucosal barrier.

In addition, guanidinoacetic acid and creatine were positively correlated with Lachnoclostridium, which is an important butyric acid-producing bacteria (Gutierrez and Garrido, 2019). Butyric acid provides energy to the gut. Liver injury leads to disturbance of energy metabolism, which recovers after treatment. It is speculated that SGD increases the level of butyric acid in the gut by regulating Lachnoclostridium. Butyric acid provides energy to the gut and promotes the synthesis of guanidinoacetic acid and creatine, thereby improving energy metabolism. Incidentally, due to the limitations of NMR, it is not possible to cover all types of metabolites, so further analysis with more techniques is required.

### 4.4 Effects of SGD on SCFAs and their correlation with gut microbiota in CCl_4_-induced liver injury

It has been concluded from previous analyses that SCFAs are associated with the metabolism of a variety of gut microbes and have important implications for the development of liver injury and the treatment of SGD. Therefore, we used GC-MS to analyze SCFAs in the cecum to reveal the changes of SCFAs during liver injury and the regulation of SGD on them. Butyric acid, a major product of gut microbial fermentation, can suppress inflammation and promote gut integrity (Tajik et al., 2020). Indeed, butyric acid also provides energy for colon cells to maintain gut health (Zhang et al., 2021c). The correlation analysis results were consistent with those presented in previous analyses. Therefore, it is speculated that SGD has the effect of restoring SCFAs and modulating the gut microbiota. Moreover, the regulation of these factors may further improve inflammation, intestinal barrier and energy metabolism.

It is worth mentioning that both fecal metabolomics and SCFAs analysis showed more severe disturbances in PGF rats, which proved the importance of gut microbiota in maintaining intestinal homeostasis. The results of PSGDT demonstrate that SGD may treat liver injury by reversing disturbed gut microbiota and its metabolites and SCFAs.

## 5 Conclusion

This study demonstrates that CCl_4_-induced liver injury increased oxidative stress, reduced antioxidant enzymes, and enhanced inflammation, which was recovered after SGD treatment. The role of gut microbiota in the pathogenesis of liver injury and the therapeutic mechanism of SGD were further investigated by 16S rRNA gene sequencing, metabolomics and SCFAs analysis. The results showed that liver injury destroyed the interrelationship of gut microbiota and disrupted ammonia transport, urea cycle, intestinal barrier and energy metabolism. Furthermore, the levels of SCFAs and the abundances of Lachnoclostridium, Blautia, Lachnospiraceae_NK4A136_group, UCG-005 and Turicibacter associated with SCFAs were also altered. However, it was reversed after SGD treatment. In addition, the analysis of PGF rats demonstrated that the gut microbiota had a protective effect on CCl_4_-induced liver injury and the efficacy of SGD might be related to the gut microbiota. In conclusion, we profiled the disturbed gut microbiota in liver injury and its association with fecal metabolites and SCFAs, and demonstrated the regulatory role of SGD on these. From the perspective of gut microbiota, it provides valuable clues for studying liver injury and the therapeutic mechanism of SGD. However, for fecal metabolomic analysis, information on all metabolites cannot be obtained using ^1^H NMR alone. In addition, 16S rRNA is more concerned with the composition of microorganisms and can only predict but not directly analyze biological functions. Therefore, more techniques are needed to further study the regulation of SGD on gut microbiota and fecal metabolites.

## Data Availability

The raw data of this study can be found in NCBI using accession number PRJNA826698. The data generated in this article are included in the article/Supplementary Materials. For further inquiries, please contact the corresponding authors directly.

## Abbreviations

ALB, albumin; ALT, Alanine aminotransferase; AST, Aspartate aminotransferase; BC, betweenness centrality; CC, Closeness centrality; Con, Control group; CYP2E1, Cytochrome P450 2E1; DC, Degree centrality; D2O, deuteroxide; GSH, Glutathione; IL-6, Interleukin six; KEGG, Kyoto Encyclopedia of Genes and Genomes; LIM, Liver injury model group; MDA, Malondialdehyde; NMDS, Non-metric multidimensional scaling analysis; PCA, Principal Component Analysis; PCF, Propyl chloroformate; PCoA, Principal co-ordinates analysis; PGF, Pseudo germ-free; PLIM, Pseudo germ-free liver injury model group; PSGDT, Pseudo germ-free SGD treatment group; SCFAs, Short-chain fatty acids; SGD, Shaoyao Gancao Decoction; SGDT, SGD treatment group; SOD, Superoxide dismutase; TNF-α, Tumor necrosis factorα; TP, total protein; TSP, Sodium 3trimethylsilyl-propionate [2,2,3,3,d4]; UPGMA, Unweighted Pair-group Method with Arithmetic Mean; VIP, Variable importance plot.

## References

[B1] Albillos A. de Gottardi A. Rescigno M. (2020). The gut-liver axis in liver disease: Pathophysiological basis for therapy. J. Hepatol. 72 (3), 558–577. 10.1016/j.jhep.2019.10.003 31622696

[B2] An X. Bao Q. Di S. Zhao Y. Zhao S. Zhang H. (2019). The interaction between the gut Microbiota and herbal medicines. Biomed. Pharmacother. 118, 109252. 10.1016/j.biopha.2019.109252 31545247

[B3] Arquero Avilès R. Marco Cuenca G. Cobo Serrano S. Ramos Simón L. F. (2014). Comunidades de práctica e innovación: Aprender a emprender en el área de Bibliotecología y ciencias de la Documentación. Investig. Bibl. Arch. Bibl. Inf. 28 (63), 193–222. 10.1016/s0187-358x(14)72580-8

[B4] Bi X. Gong M. Di L. (2014). Review on prescription compatibility of shaoyao gancao decoction and reflection on pharmacokinetic compatibility mechanism of traditional Chinese medicine prescription based on *in vivo* drug interaction of main efficacious components. Evid. Based. Complement. Altern. Med. 2014, 208129. 10.1155/2014/208129 PMC4132488 25147573

[B5] Campana L. Iredale J. P. (2017). Regression of liver fibrosis. Semin. Liver Dis. 37 (1), 1–10. 10.1055/s-0036-1597816 28201843

[B6] Chen J. Kang B. Jiang Q. Han M. Zhao Y. Long L. (2018). Alpha-ketoglutarate in low-protein diets for growing pigs: Effects on cecal microbial communities and parameters of microbial metabolism. Front. Microbiol. 9, 1057. 10.3389/fmicb.2018.01057 29904374 PMC5991137

[B7] Chen J. Vitetta L. (2020). Gut microbiota metabolites in NAFLD pathogenesis and therapeutic implications. Int. J. Mol. Sci. 21 (15), E5214. 10.3390/ijms21155214 32717871 PMC7432372

[B8] Fung T. C. Vuong H. E. Luna C. D. G. Pronovost G. N. Aleksandrova A. A. Riley N. G. (2019). Intestinal serotonin and fluoxetine exposure modulate bacterial colonization in the gut. Nat. Microbiol. 4 (12), 2064–2073. 10.1038/s41564-019-0540-4 31477894 PMC6879823

[B9] Gu X. Manautou J. E. (2012). Molecular mechanisms underlying chemical liver injury. Expert Rev. Mol. Med. 14, e4. 10.1017/S1462399411002110 22306029 PMC3704158

[B10] Guo C. Li Y. Wang P. Li Y. Qiu C. Li M. (2018). Alterations of gut microbiota in cholestatic infants and their correlation with hepatic function. Front. Microbiol. 9, 2682. 10.3389/fmicb.2018.02682 30483228 PMC6243132

[B11] Gupta S. Fenves A. Z. Hootkins R. (2016). The role of RRT in hyperammonemic patients. Clin. J. Am. Soc. Nephrol. 11 (10), 1872–1878. 10.2215/CJN.01320216 27197910 PMC5053785

[B12] Gutierrez N. Garrido D. (2019). Species deletions from microbiome consortia reveal key metabolic interactions between gut microbes. mSystems 4 (4), e00185–19. 10.1128/mSystems.00185-19 31311843 PMC6635622

[B13] He G. Karin M. (2011). NF-κB and STAT3 - key players in liver inflammation and cancer. Cell Res. 21 (1), 159–168. 10.1038/cr.2010.183 21187858 PMC3193410

[B14] Herp S. Durai Raj A. C. Salvado Silva M. Woelfel S. Stecher B. (2021). The human symbiont Mucispirillum schaedleri: Causality in health and disease. Med. Microbiol. Immunol. 210 (4), 173–179. 10.1007/s00430-021-00702-9 34021796 PMC7615636

[B15] Jones R. M. Neish A. S. (2021). Gut microbiota in intestinal and liver disease. Annu. Rev. Pathol. 16, 251–275. 10.1146/annurev-pathol-030320-095722 33234022

[B16] Kamimura K. Inoue R. Nagoya T. Sakai N. Goto R. Ko M. (2018). Autonomic nervous system network and liver regeneration. World J. Gastroenterol. 24 (15), 1616–1621. 10.3748/wjg.v24.i15.1616 29686468 PMC5910544

[B17] Khan I. Huang Z. Liang L. Li N. Ali Z. Ding L. (2021). Ammonia stress influences intestinal histomorphology, immune status and microbiota of Chinese striped-neck turtle (Mauremys sinensis). Ecotoxicol. Environ. Saf. 222, 112471. 10.1016/j.ecoenv.2021.112471 34229168

[B18] Kim C. H. Park J. Kim M. (2014). Gut microbiota-derived short-chain Fatty acids, T cells, and inflammation. Immune Netw. 14 (6), 277–288. 10.4110/in.2014.14.6.277 25550694 PMC4275385

[B19] Kim J. Y. Han Y. H. Nam M. W. Kim H. J. Lee M. O. (2019). RORα suppresses interleukin-6-mediated hepatic acute phase response. Sci. Rep. 9 (1), 11798. 10.1038/s41598-019-48171-8 31409825 PMC6692401

[B20] Kim M. H. Kim H. (2017). The roles of glutamine in the intestine and its implication in intestinal diseases. Int. J. Mol. Sci. 18 (5), E1051. 10.3390/ijms18051051 28498331 PMC5454963

[B21] Koh G. Y. Kane A. V. Wu X. Crott J. W. (2020). Parabacteroides distasonis attenuates tumorigenesis, modulates inflammatory markers and promotes intestinal barrier integrity in azoxymethane-treated A/J mice. Carcinogenesis 41 (7), 909–917. 10.1093/carcin/bgaa018 32115637

[B22] Kong C. Gao R. Yan X. Huang L. Qin H. (2019). Probiotics improve gut microbiota dysbiosis in obese mice fed a high-fat or high-sucrose diet. Nutrition 60, 175–184. 10.1016/j.nut.2018.10.002 30611080

[B23] Kong L. Chen J. Ji X. Qin Q. Yang H. Liu D. (2021). Alcoholic fatty liver disease inhibited the co-expression of Fmo5 and PPARα to activate the NF-κB signaling pathway, thereby reducing liver injury via inducing gut microbiota disturbance. J. Exp. Clin. Cancer Res. 40 (1), 18. 10.1186/s13046-020-01782-w 33413501 PMC7788704

[B24] Kwo P. Y. Cohen S. M. Lim J. K. (2017). ACG clinical guideline: Evaluation of abnormal liver chemistries. Am. J. Gastroenterol. 112 (1), 18–35. 10.1038/ajg.2016.517 27995906

[B25] Leung C. Rivera L. Furness J. B. Angus P. W. (2016). The role of the gut microbiota in NAFLD. Nat. Rev. Gastroenterol. Hepatol. 13 (7), 412–425. 10.1038/nrgastro.2016.85 27273168

[B26] Levy M. Blacher E. Elinav E. (2017). Microbiome, metabolites and host immunity. Curr. Opin. Microbiol. 35, 8–15. 10.1016/j.mib.2016.10.003 27883933

[B27] Li T. Chiang J. Y. (2014). Bile acid signaling in metabolic disease and drug therapy. Pharmacol. Rev. 66 (4), 948–983. 10.1124/pr.113.008201 25073467 PMC4180336

[B28] Li Y. Hu H. Yang H. Lin A. Xia H. Cheng X. (2022). Vine tea (ampelopsis grossedentata) extract attenuates CCl4 -induced liver injury by restoring gut microbiota dysbiosis in mice. Mol. Nutr. Food Res. 66, e2100892. 10.1002/mnfr.202100892 35188709

[B29] Liu X. Mao B. Gu J. Wu J. Cui S. Wang G. (2021). Blautia-a new functional genus with potential probiotic properties? Gut Microbes 13 (1), 1–21. 10.1080/19490976.2021.1875796 PMC7872077 33525961

[B30] Liu Z. Wang X. Li L. Wei G. Zhao M. (2020). Hydrogen sulfide protects against paraquat-induced acute liver injury in rats by regulating oxidative stress, mitochondrial function, and inflammation. Oxid. Med. Cell. Longev. 2020, 6325378. 10.1155/2020/6325378 32064027 PMC6998754

[B31] Ma L. Ni Y. Wang Z. Tu W. Ni L. Zhuge F. (2020). Spermidine improves gut barrier integrity and gut microbiota function in diet-induced obese mice. Gut Microbes 12 (1), 1–19. 10.1080/19490976.2020.1832857 PMC7668533 33151120

[B32] Mangifesta M. Mancabelli L. Milani C. Gaiani F. de'Angelis N. de'Angelis G. L. (2018). Mucosal microbiota of intestinal polyps reveals putative biomarkers of colorectal cancer. Sci. Rep. 8 (1), 13974. 10.1038/s41598-018-32413-2 30228361 PMC6143603

[B33] Martin-Gallausiaux C. Marinelli L. Blottiere H. M. Larraufie P. Lapaque N. S. C. F. A. (2021). Scfa: Mechanisms and functional importance in the gut. Proc. Nutr. Soc. 80 (1), 37–49. 10.1017/S0029665120006916 32238208

[B34] McCarty M. F. Lerner A. (2021). Perspective: Prospects for nutraceutical support of intestinal barrier function. Adv. Nutr. 12 (2), 316–324. 10.1093/advances/nmaa139 33126251 PMC8243597

[B35] Michalopoulos G. K. (2017). Hepatostat: Liver regeneration and normal liver tissue maintenance. Hepatology 65 (4), 1384–1392. 27997988 10.1002/hep.28988

[B36] Nagao-Kitamoto H. Leslie J. L. Kitamoto S. Jin C. Thomsson K. A. GillillandKuffa M. G. P. 3rd (2020). Interleukin-22-mediated host glycosylation prevents Clostridioides difficile infection by modulating the metabolic activity of the gut microbiota. Nat. Med. 26 (4), 608–617. 10.1038/s41591-020-0764-0 32066975 PMC7160049

[B37] Nagayama M. Yano T. Atarashi K. Tanoue T. Sekiya M. Kobayashi Y. (2020). TH1 cell-inducing *Escherichia coli* strain identified from the small intestinal mucosa of patients with Crohn's disease. Gut Microbes 12 (1), 1788898. 10.1080/19490976.2020.1788898 PMC7524366 32691669

[B38] Rahman M. M. Shahab N. B. Miah P. Rahaman M. M. Kabir A. U. Subhan N. (2021). Polyphenol-rich leaf of Aphanamixis polystachya averts liver inflammation, fibrogenesis and oxidative stress in ovariectomized Long-Evans rats. Biomed. Pharmacother. 138, 111530. 10.1016/j.biopha.2021.111530 33773464

[B39] Rhoads J. M. Chen W. Gookin J. Wu G. Y. Fu Q. Blikslager A. T. (2004). Arginine stimulates intestinal cell migration through a focal adhesion kinase dependent mechanism. Gut 53 (4), 514–522. 10.1136/gut.2003.027540 15016745 PMC1774018

[B40] Richard M. L. Liguori G. Lamas B. Brandi G. da Costa G. Hoffmann T. W. (2018). Mucosa-associated microbiota dysbiosis in colitis associated cancer. Gut Microbes 9 (2), 131–142. 10.1080/19490976.2017.1379637 28914591 PMC5989788

[B41] Schmidt-Arras D. Rose-John S. (2016). IL-6 pathway in the liver: From physiopathology to therapy. J. Hepatol. 64 (6), 1403–1415. 10.1016/j.jhep.2016.02.004 26867490

[B42] Schoeler M. Caesar R. (2019). Dietary lipids, gut microbiota and lipid metabolism. Rev. Endocr. Metab. Disord. 20 (4), 461–472. 10.1007/s11154-019-09512-0 31707624 PMC6938793

[B43] Shang Q. H. Liu S. J. He T. F. Liu H. S. Mahfuz S. Ma X. K. (2020). Effects of wheat bran in comparison to antibiotics on growth performance, intestinal immunity, barrier function, and microbial composition in broiler chickens. Poult. Sci. 99 (10), 4929–4938. 10.1016/j.psj.2020.06.031 32988529 PMC7598142

[B44] Shi W. Shen L. Zou W. Wang J. Yang J. Wang Y. (2020). The gut microbiome is associated with therapeutic responses and toxicities of neoadjuvant chemoradiotherapy in rectal cancer patients-A pilot study. Front. Cell. Infect. Microbiol. 10, 562463. 10.3389/fcimb.2020.562463 33363048 PMC7756020

[B45] Sun L. Zhao M. Zhao Y. h. Jiang X. Wang M. Zhang Y. x. (2020). Rapid characterization of chemical constituents of Shaoyao Gancao decoction using UHPLC coupled with Fourier transform ion cyclotron resonance mass spectrometry. RSC Adv. 10 (49), 29528–29535. 10.1039/d0ra04701e 35521121 PMC9055985

[B46] Sun L. Zhao M. Zhao Y. Wang M. Man J. Zhao C. (2020). Investigation of the therapeutic effect of Shaoyao Gancao decoction on CCL4 -induced liver injury in rats by metabolomic analysis. Biomed. Chromatogr. 34 (11), e4940. 10.1002/bmc.4940 32634249

[B47] Tajik N. Frech M. Schulz O. Schalter F. Lucas S. Azizov V. (2020). Targeting zonulin and intestinal epithelial barrier function to prevent onset of arthritis. Nat. Commun. 11 (1), 1995. 10.1038/s41467-020-15831-7 32332732 PMC7181728

[B48] Tilg H. Cani P. D. Mayer E. A. (2016). Gut microbiome and liver diseases. Gut 65 (12), 2035–2044. 10.1136/gutjnl-2016-312729 27802157

[B49] Tilg H. Zmora N. Adolph T. E. Elinav E. (2020). The intestinal microbiota fuelling metabolic inflammation. Nat. Rev. Immunol. 20 (1), 40–54. 10.1038/s41577-019-0198-4 31388093

[B50] Traussnigg S. Kienbacher C. Gajdosik M. Valkovic L. Halilbasic E. Stift J. (2017). Ultra-high-field magnetic resonance spectroscopy in non-alcoholic fatty liver disease: Novel mechanistic and diagnostic insights of energy metabolism in non-alcoholic steatohepatitis and advanced fibrosis. Liver Int. 37 (10), 1544–1553. 10.1111/liv.13451 28544208 PMC5638103

[B51] Tsikas D. (2017). Assessment of lipid peroxidation by measuring malondialdehyde (MDA) and relatives in biological samples: Analytical and biological challenges. Anal. Biochem. 524, 13–30. 10.1016/j.ab.2016.10.021 27789233

[B52] Unsal V. Cicek M. Sabancilar I. (2021). Toxicity of carbon tetrachloride, free radicals and role of antioxidants. Rev. Environ. Health 36 (2), 279–295. 10.1515/reveh-2020-0048 32970608

[B53] Wang C. Ma C. Fu K. Gong L. H. Zhang Y. F. Zhou H. L. (2021). Phillygenin attenuates carbon tetrachloride-induced liver fibrosis via modulating inflammation and gut microbiota. Front. Pharmacol. 12, 756924. 10.3389/fphar.2021.756924 34621179 PMC8490881

[B54] Wang K. Liao M. Zhou N. Bao L. Ma K. Zheng Z. (2019). Parabacteroides distasonis alleviates obesity and metabolic dysfunctions via production of succinate and secondary bile acids. Cell Rep. 26 (1), 222–235. 10.1016/j.celrep.2018.12.028 30605678

[B55] Wang X. Qiao S. Yin Y. Yue L. Wang Z. Wu G. (2007). A deficiency or excess of dietary threonine reduces protein synthesis in jejunum and skeletal muscle of young pigs. J. Nutr. 137 (6), 1442–1446. 10.1093/jn/137.6.1442 17513404

[B56] Wolfarth A. A. Liu X. Darby T. M. Boyer D. J. Spizman J. B. Owens J. A. (2020). Proline-Rich acidic protein 1 (PRAP1) protects the gastrointestinal epithelium from irradiation-induced apoptosis. Cell. Mol. Gastroenterol. Hepatol. 10 (4), 713–727. 10.1016/j.jcmgh.2020.06.011 32629119 PMC7498829

[B57] Xiao S. Liu C. Chen M. Zou J. Zhang Z. Cui X. (2020). Scutellariae radix and coptidis rhizoma ameliorate glycolipid metabolism of type 2 diabetic rats by modulating gut microbiota and its metabolites. Appl. Microbiol. Biotechnol. 104 (1), 303–317. 10.1007/s00253-019-10174-w 31758238

[B58] Yao Y. Yan L. Chen H. Wu N. Wang W. Wang D. (2020). Cyclocarya paliurus polysaccharides alleviate type 2 diabetic symptoms by modulating gut microbiota and short-chain fatty acids. Phytomedicine 77, 153268. 10.1016/j.phymed.2020.153268 32663709

[B59] Zhang C. Zhao J. Famous E. Pan S. Peng X. Tian J. (2021). Antioxidant, hepatoprotective and antifungal activities of black pepper (Piper nigrum L.) essential oil. Food Chem. 346, 128845. 10.1016/j.foodchem.2020.128845 33387832

[B60] Zhang J. Wang Z. Huo D. Shao Y. (2018). Consumption of goats' milk protects mice from carbon tetrachloride-induced acute hepatic injury and improves the associated gut microbiota imbalance. Front. Immunol. 9, 1034. 10.3389/fimmu.2018.01034 29867999 PMC5962680

[B61] Zhang L. Liu C. Jiang Q. Yin Y. (2021). Butyrate in energy metabolism: There is still more to learn. Trends Endocrinol. Metab. 32 (3), 159–169. 10.1016/j.tem.2020.12.003 33461886

[B62] Zhang Y. Zhao M. Jiang X. Qiao Q. Liu T. Zhao C. (2021). Comprehensive analysis of fecal microbiome and metabolomics in hepatic fibrosis rats reveal hepatoprotective effects of yinchen wuling powder from the host-microbial metabolic Axis. Front. Pharmacol. 12, 713197. 10.3389/fphar.2021.713197 34385924 PMC8353151

[B63] Zhao Z. H. Xin F. Z. Xue Y. Hu Z. Han Y. Ma F. (2019). Indole-3-propionic acid inhibits gut dysbiosis and endotoxin leakage to attenuate steatohepatitis in rats. Exp. Mol. Med. 51 (9), 1–14. 10.1038/s12276-019-0304-5 PMC6802644 31506421

[B64] Zheng J. Hoffman K. L. Chen J. S. Shivappa N. Sood A. Browman G. J. (2020). Dietary inflammatory potential in relation to the gut microbiome: Results from a cross-sectional study. Br. J. Nutr. 124 (9), 931–942. 10.1017/S0007114520001853 32475373 PMC7554089

