# Supplementary material for: Combining fecal microbiome and metabolomics to reveal the disturbance of gut microbiota in liver injury and the therapeutic mechanism of shaoyao gancao decoction
Source: Front Pharmacol. 2022 Aug 16;13:911356. doi: 10.3389/fphar.2022.911356 (PMC9428823; doi:10.3389/fphar.2022.911356)
Supplement: Supplementary file 1 [file DataSheet1.docx]

***Supplementary material***

**1 Supplementary Data**

**1.1 Sample preparation**

100 mg of cecum contents were added to 1 mL of 0.005 M aqueous NaOH along with the internal standard (IS, crotonic acid, final concentration of 10 µg/mL). Vortex for 2 min and centrifuge at 13000 rpm for 10 min. Take 500 µL supernatant and add 300 µL n-propanol, 200 µL pyridine and 100 µL propyl chloroformate (PCF). Open the cover for 1 min to discharge the generated gas, and swirl for 1 min. Add 300 µL n-hexane, vortex for 1 min, and centrifuge at 10000 rpm for 5 min. Transfer the n-hexane layer to the sample bottle.

**1.2 Instrument conditions**

GC-MS of Thermo Trace 1300-ISQ (Thermo Fisher, USA) with TG-5MS (30 m×0.25 mm, 0.25 μm) capillary column was used to determine the content of SCFAs. Helium was used as carrier gas, and 1ul derivative was injected into the sample in a 10: 1 ratio at a flow rate of 1 mL/min. The temperatures of the inlet, transmission line and ion source were 260℃, 290℃ and 300℃ respectively. The initial column temperature was maintained at 30℃ for 2min, rising from 10℃/min to 45℃ for 10min, and rising from 30℃/min to 200℃ for 3min. The solvent delay time was 3min. Electron bombardment ion source (EI) was used, and the electron energy was -70 eV. Mass spectrum data were obtained in full scanning mode (m/z 30-200). Quantitative analysis was carried out in SIM scanning mode. The quantitative ions were shown in Supplementary Table S1. Data processing was performed in Thermo Xcalibur (4.1.31.9) Qual Browser and Quan Browser. Detailed procedures for the preparation of standard solutions and methodological validation were provided in Supplementary Materials.

**1.3 Solution preparation**

The seven SCFAs were precisely weighed and dissolved in 0.005 M aqueous NaOH to prepare a mixed standard stock solution containing acetic acid (1 mg/ml), propionic acid (1 mg/ml), isobutyric acid (0.1 mg/ml), butyric acid (1 mg/ml), isovaleric acid (0.1 mg/ml), valeric acid (0.1 mg/ml), and caproic acid (0.1 mg/ml). Crotonic acid stock solutions (1 mg/ml) were also prepared with 0.005M aqueous NaOH for internal standard (IS). Serial dilutions were made with 0.005 M aqueous NaOH to prepare a series of mixed standard solutions with the addition of IS (final concentration 10 µg/mL). The concentrations of acetic acid, propionic acid and butyric acid were 1, 2, 5, 10, 20, 50, 100, 200 and 500 μg/ml, and isobutyric acid, isovaleric acid, valeric acid and caproic acid were 0.1, 0.2, 0.5, 1, 2, 5, 10, 20 and 50 μg/ml. Meanwhile, the mixed standard solutions with low, medium and high concentrations were prepared as quality control (QC) samples. All standard stock solutions were stored at -20°C, while all working solutions were stored at 4°C.

**1.4 Method validation**

To ensure the reliability of the quantitative method, method validation was performed, including specificity, linearity range, precision, accuracy, recovery, matrix effects, and stability. Specificity was investigated by analyzing IS-free standards, IS-containing standards and samples. As shown in Supplementary Figure S14, the seven SCFAs and IS were well separated and not disturbed by endogenous substances. Linearity was determined from a calibration curve constructed from a series of mixed standard solutions at nine concentrations. Two-sample analysis for each concentration. Take the peak area ratio of the analyte and IS as the ordinate, and the corresponding concentration as the abscissa. Linear regression was performed with weighted least squares (1/χ^2^). Concentrations with an S/N ratio of 10 were considered to be LLOQ. The results were shown in Supplementary Table S8, and the analytes have a good linear relationship in their respective concentration ranges. The intra-day precision, inter-day precision for three consecutive days and accuracy were determined by QC samples with low, medium and high concentrations, with 6 samples for each concentration. The results were shown in Supplementary Table S9, which meet the measurement requirements. The recovery was the difference between the measured concentrations of the samples with and without QC added to the ratio of the corresponding true concentrations. Subtract the peak area of the sample with QC added after extraction from the peak area of the sample with QC added before extraction. Then divide it by the peak area of 0.005M NaOH, and the result is the matrix effect. The results were shown in Supplementary Table S9, the recovery rate meet the requirements, and the matrix did not interfere with the determination. The stability was checked by placing the QC samples at 4℃ for 24 h, three freeze-thaw cycles and storage at -80°C for 1 week. The results were shown in Supplementary Table S10, showing good stability under the above conditions.

**2 Supplementary Figures**


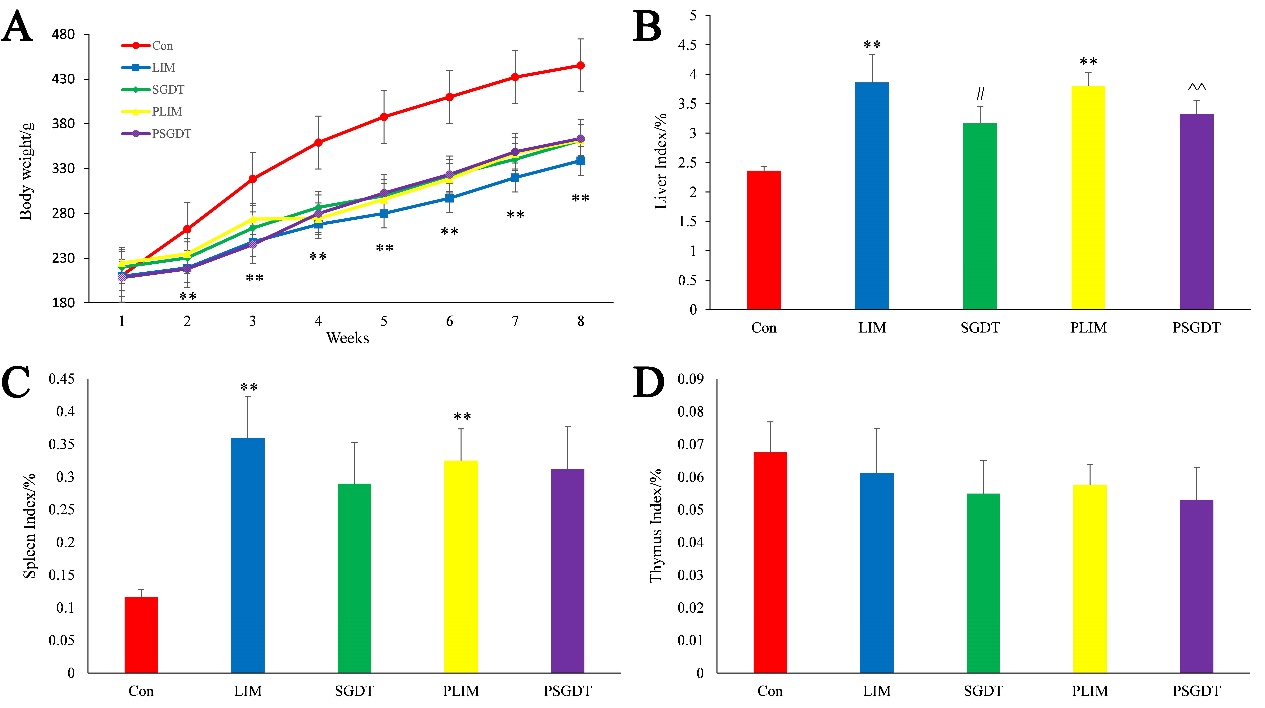


Fig. S1 Effects of SGD on body weight and organ index of rats (n=6). (A) Changes of rat body weight; (B) Liver index; (C) Spleen index; (D) Thymus index. *p＜0.05, **p＜0.01, compare with Con; # p＜0.05, compare with LIM; ^^ p＜0.01, compare with PLIM.


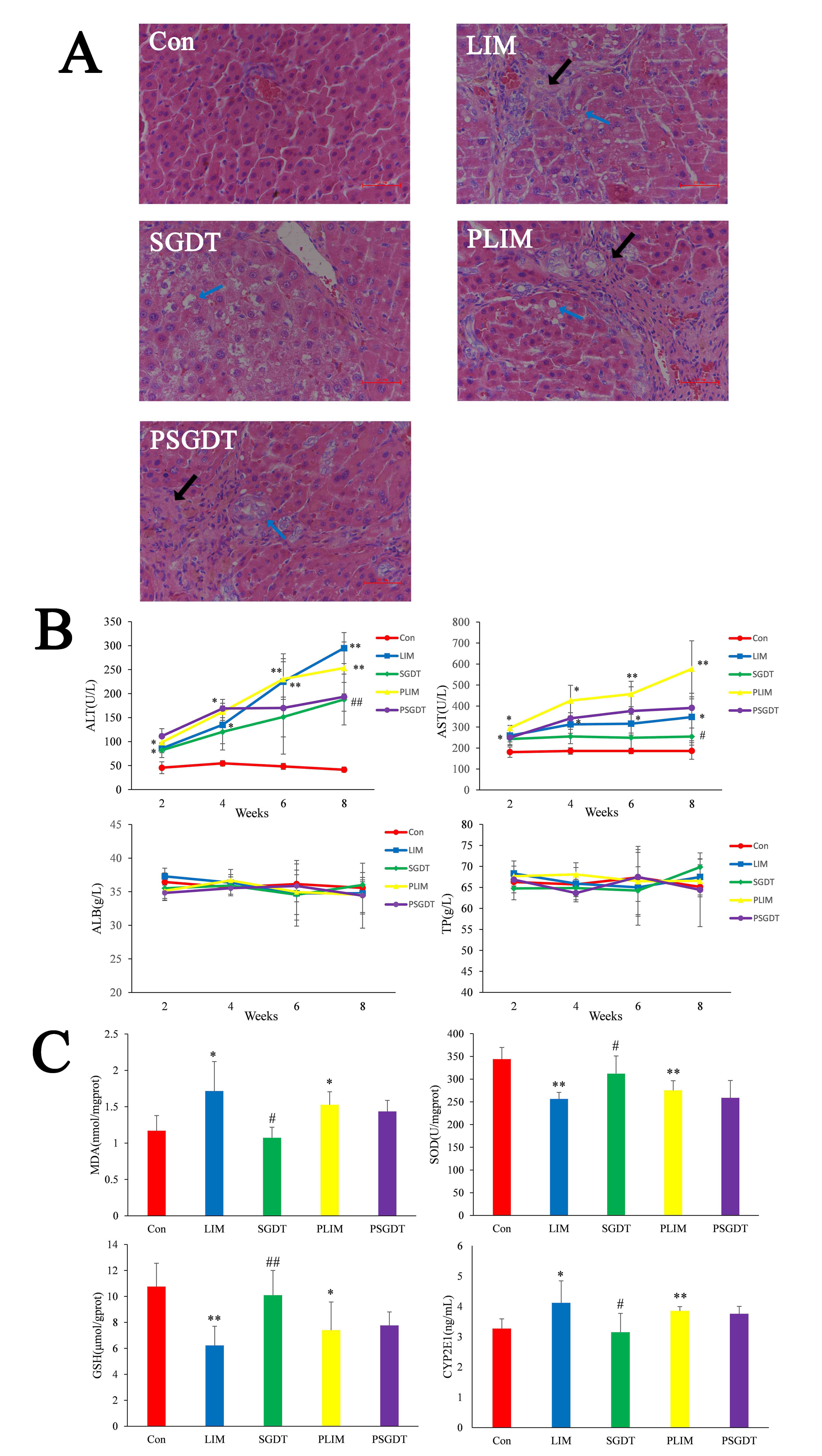


Fig. S2 Effects of SGD on CCl_4_-induced liver injury. (A) Histopathology of liver H&E (400 ×) staining in Con, LIM, SGDT, PLIM and PSGDT. Black arrows point to fibrous connective tissue hyperplasia, and blue arrows point to inflammatory cell infiltration and vacuolar degeneration. (B) During the experiment, the serum ALT, AST, ALB and TP levels of five groups. (C) The levels of MDA, SOD, GSH and CYP2E1 in the liver of five groups. *p＜0.05, **p＜0.01, compare with Con; # p＜0.05, ## p＜0.01 compare with LIM.


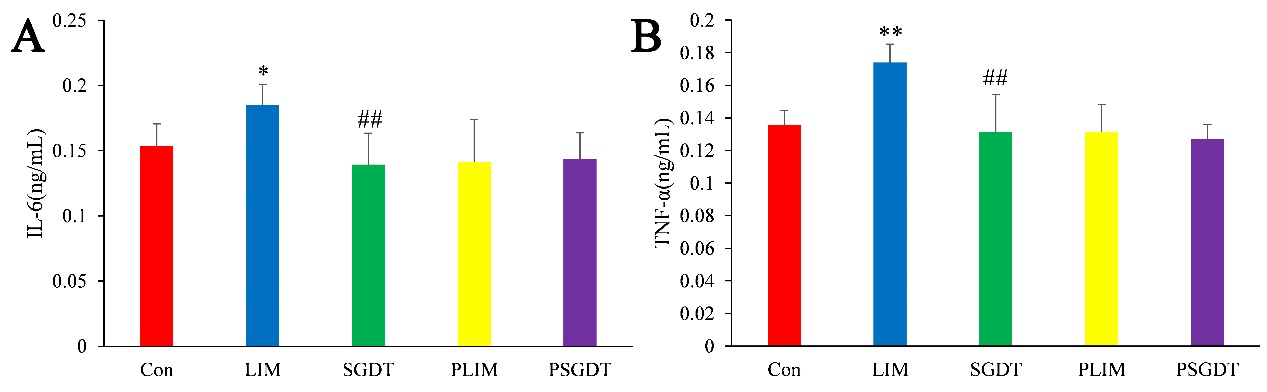


Fig. S3 Plasma inflammatory cytokine levels. (A) IL-6 (B) TNF-α. *p＜0.05, **p＜0.01, compare with Con; ## p＜0.01 compare with LIM.


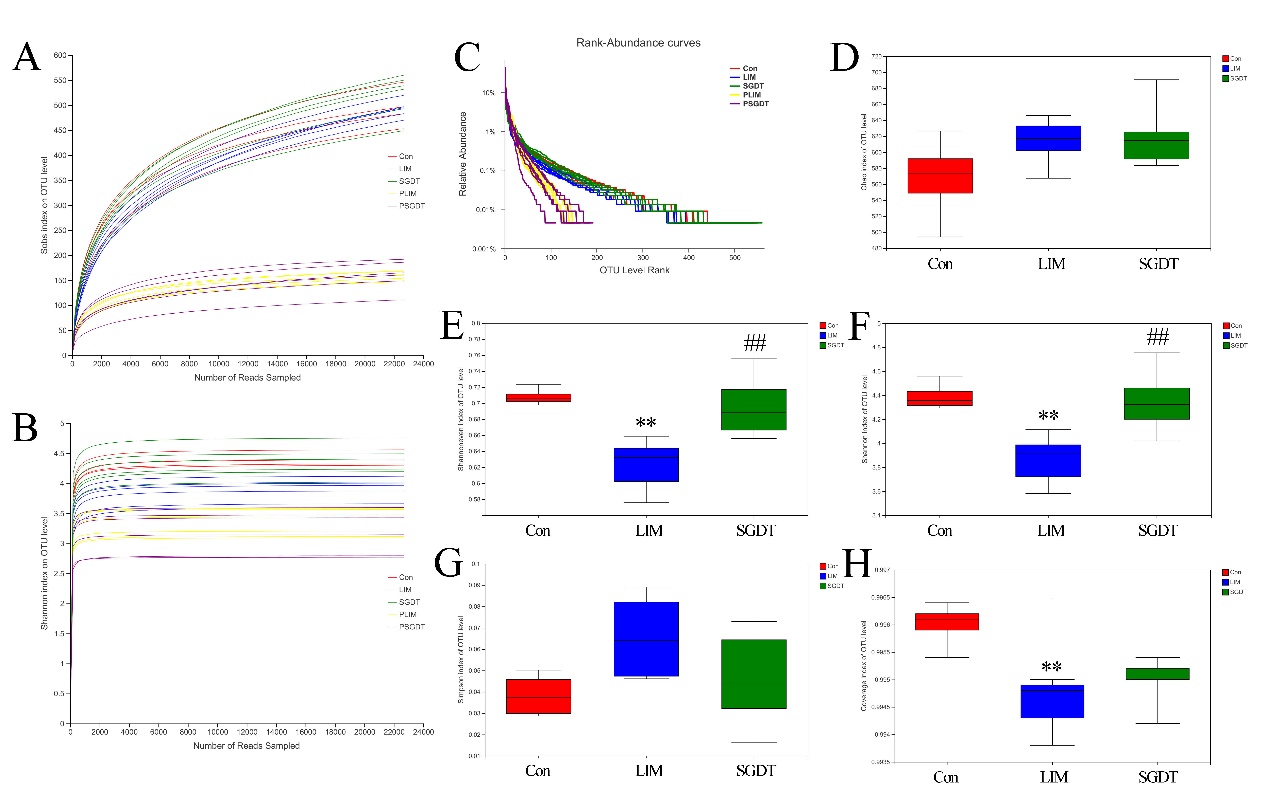


Fig. S4 Microbial annotation and assessment of 16S rRNA gene sequencing. (A, B) Rarefaction curves based on Sobs and Shannon. (C) Rank-Abundance curves. (D) Chao index. (E) Shannoneven index. (F) Shannon index. (G) Simpson index. (H) Coverage index. **p＜0.01, compare with Con; ## p＜0.01, compare with LIM.


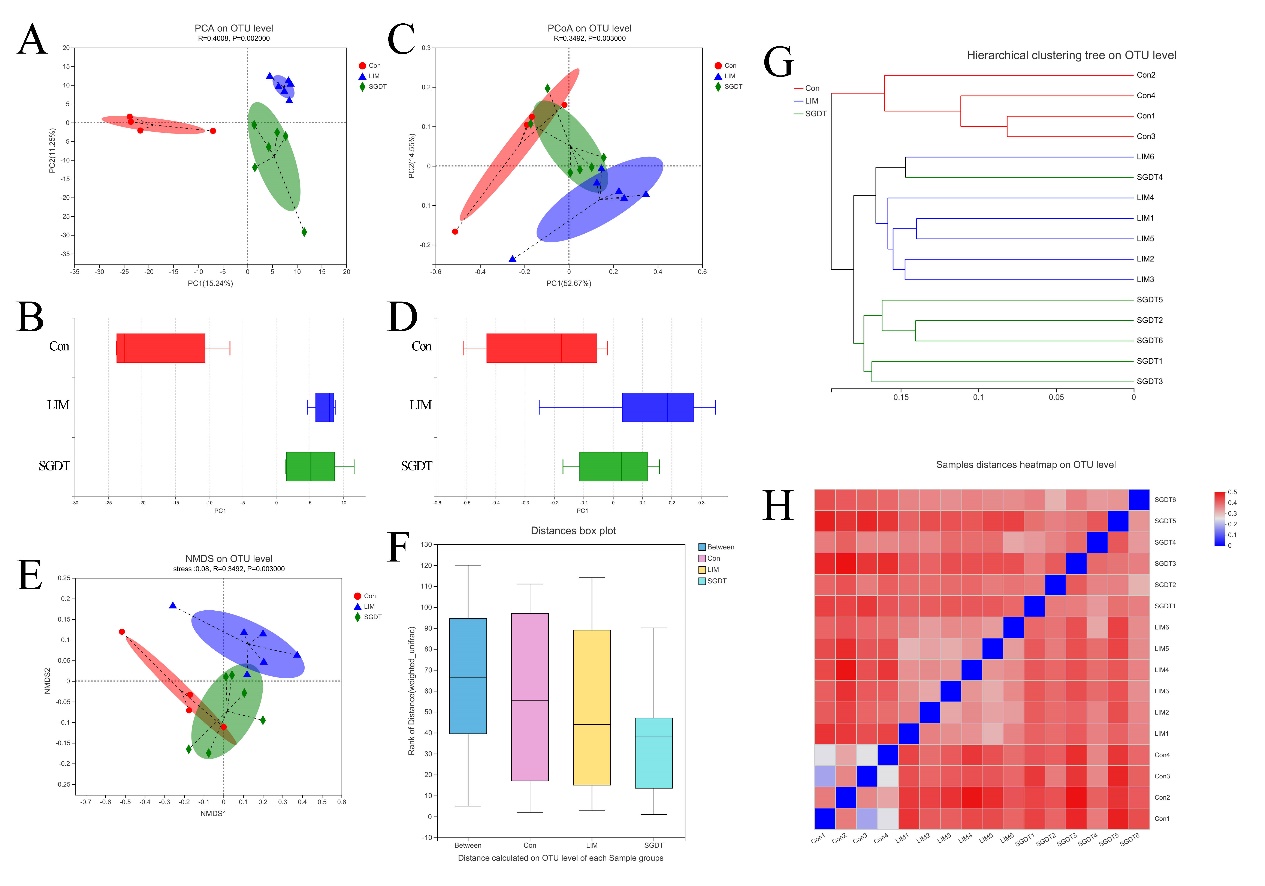


Fig. S5 Comparative analysis of microbial communities among Con, LIM and SGDT. (A) PCA analysis. (B) Distribution of different groups on PC1 axis in PCA analysis. (C) Weighted_unifrac-based PCoA analysis. (D) Distribution of different groups on PC1 axis in PCoA analysis. (E) Weighted_unifrac-based NMDS analysis. (F) ANOSIM analysis. (G) Unweighted_unifrac-based heatmap. (H) Hierarchical clustering with UPGMA based on unweighted_unifrac.


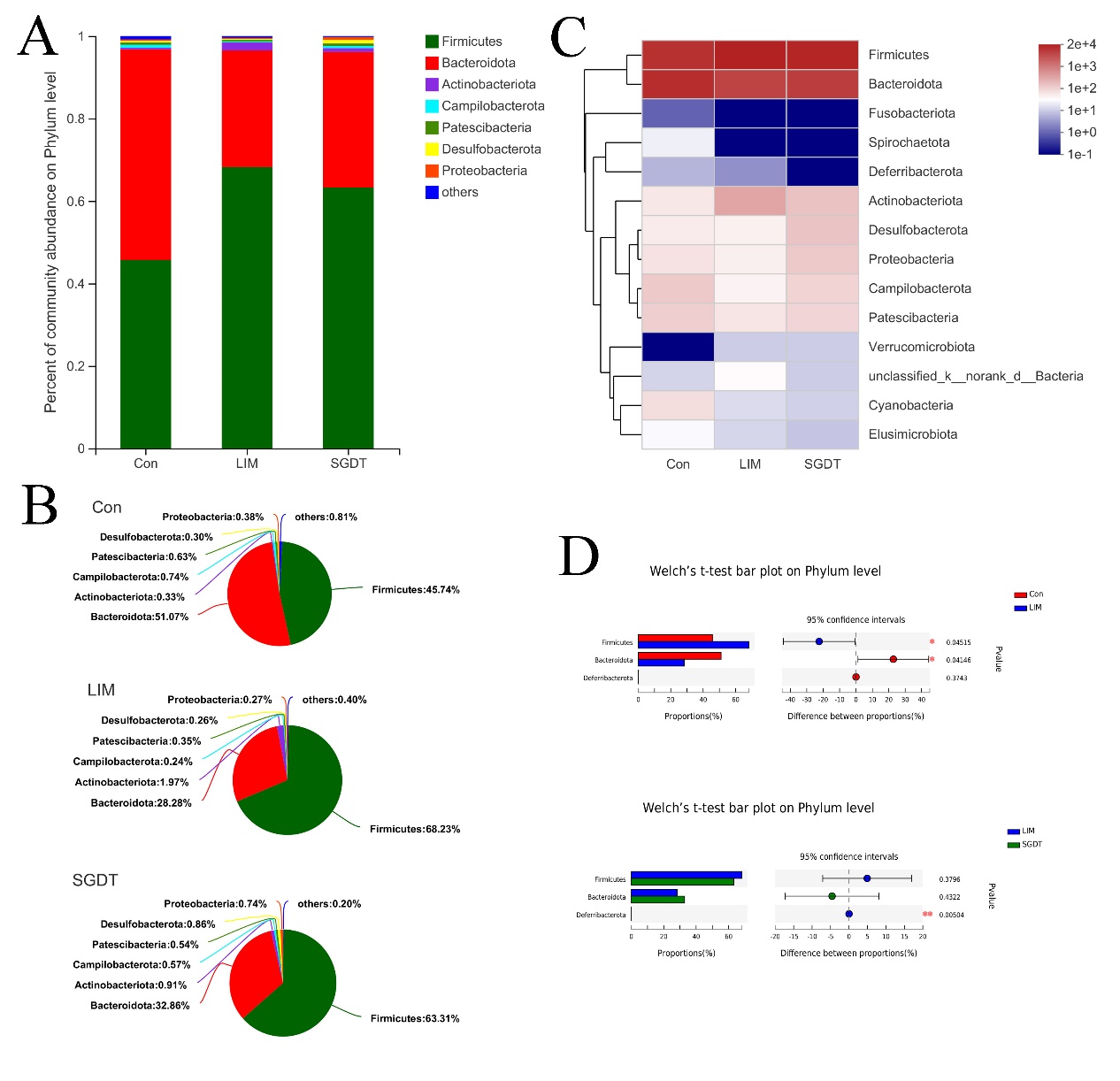


Fig. S6 The effect of SGD on the structure of gut microbiota in rats with CCl_4_-induced liver injury was analyzed by Con, LIM and SGDT at the phylum level. (A) Community bar chart. (B) Community pie chart. (C) Community heatmap. (D) Test of inter-group differences based on welch's t-test between Con and LIM and between LIM and SGDT.


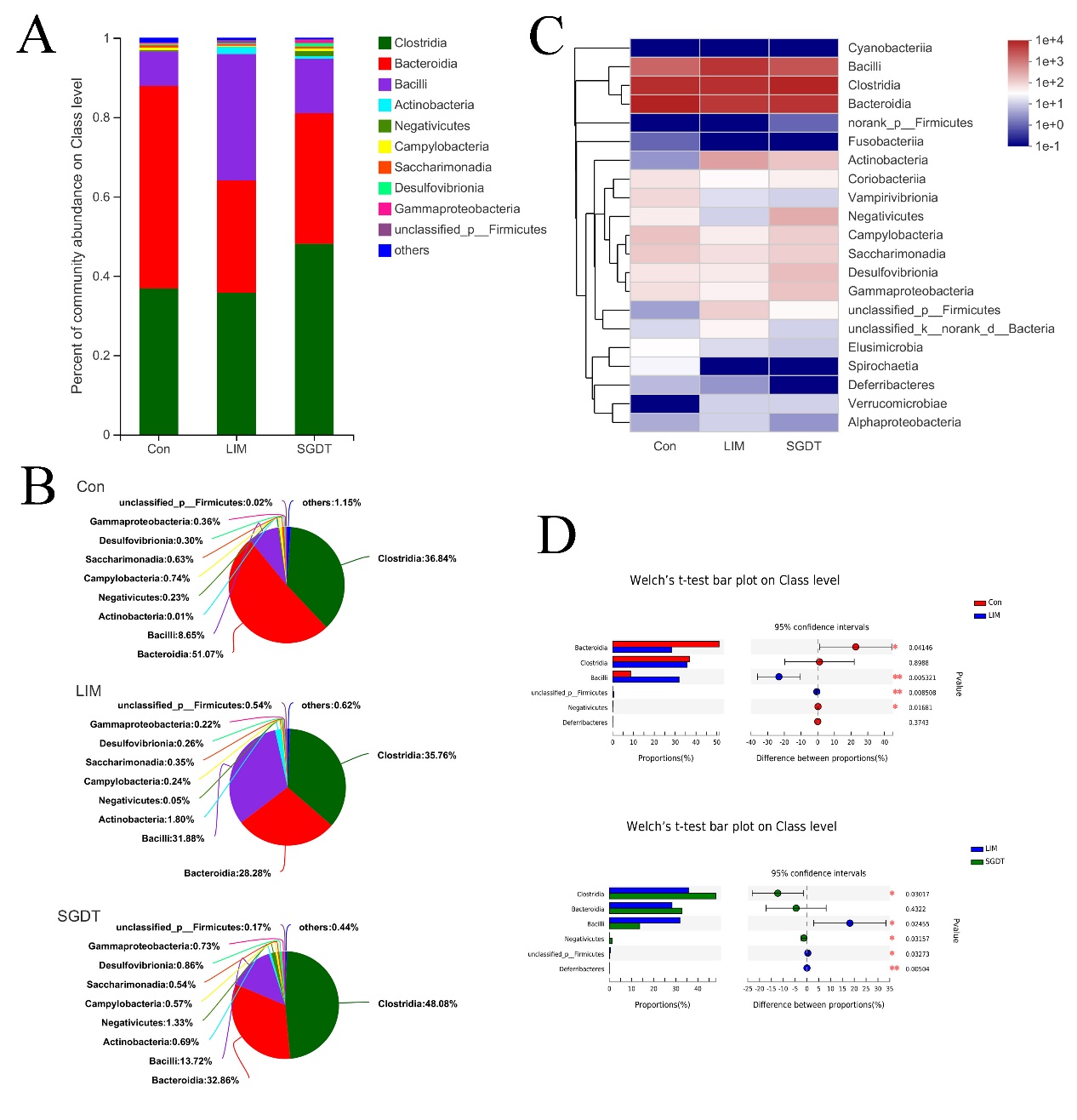


Fig. S7 The effect of SGD on the structure of gut microbiota in rats with CCl_4_-induced liver injury was analyzed by Con, LIM and SGDT at the class level. (A) Community bar chart. (B) Community pie chart. (C) Community heatmap. (D) Test of inter-group differences based on welch's t-test between Con and LIM and between LIM and SGDT.


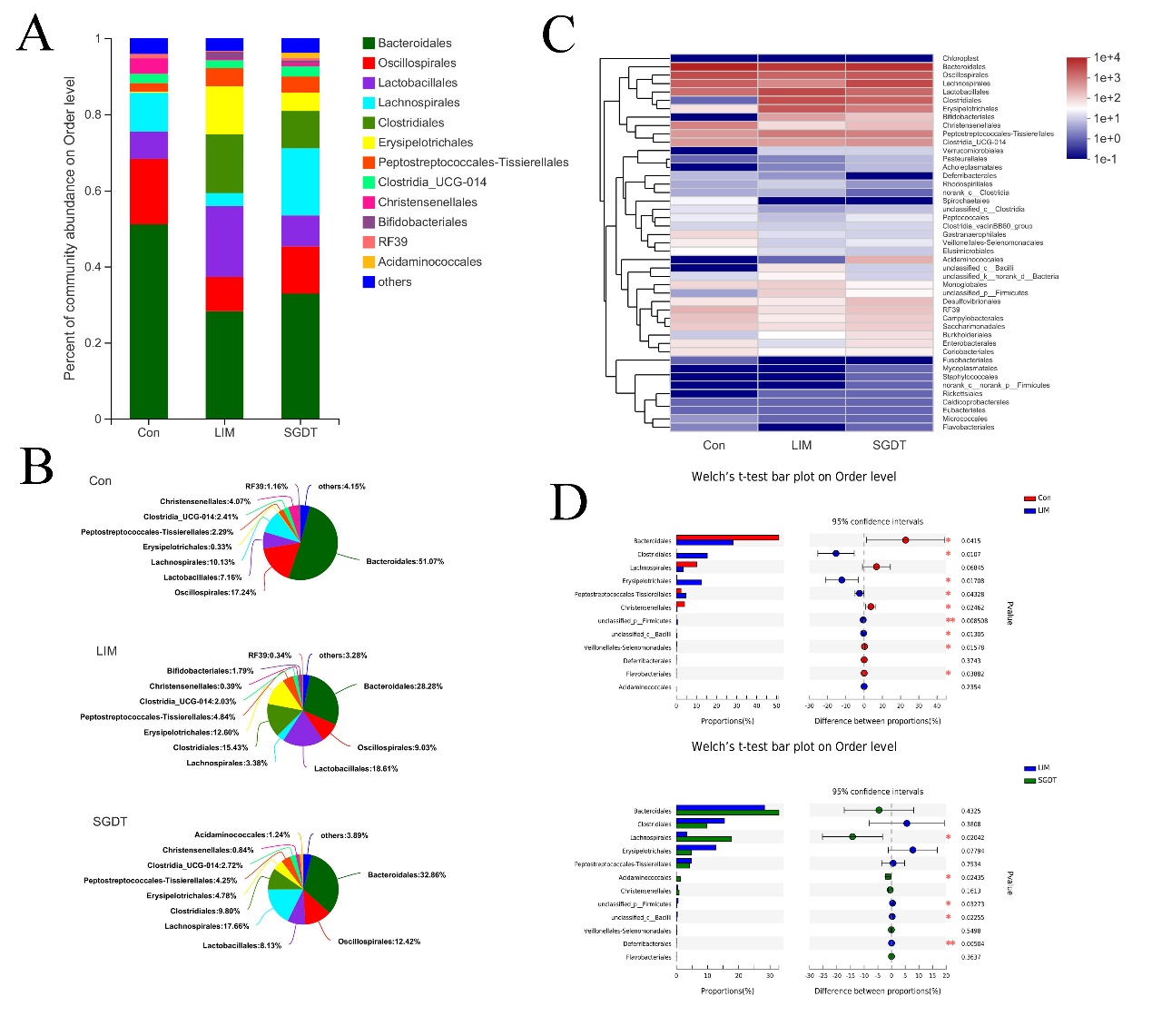


Fig. S8 The effect of SGD on the structure of gut microbiota in rats with CCl_4_-induced liver injury was analyzed by Con, LIM and SGDT at the order level. (A) Community bar chart. (B) Community pie chart. (C) Community heatmap. (D) Test of inter-group differences based on welch's t-test between Con and LIM and between LIM and SGDT.


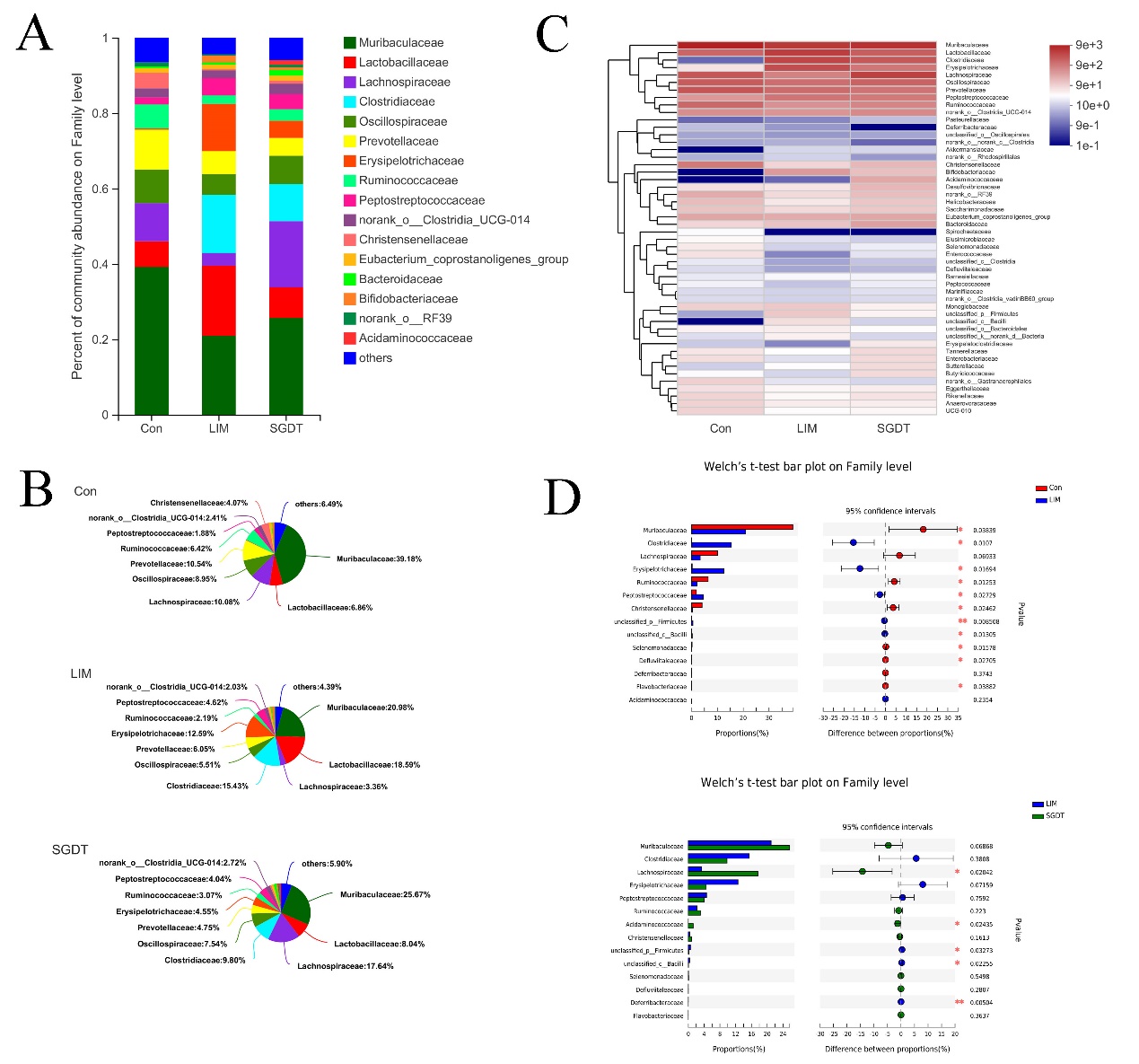


Fig. S9 The effect of SGD on the structure of gut microbiota in rats with CCl_4_-induced liver injury was analyzed by Con, LIM and SGDT at the family level. (A) Community bar chart. (B) Community pie chart. (C) Community heatmap. (D) Test of inter-group differences based on welch's t-test between Con and LIM and between LIM and SGDT.


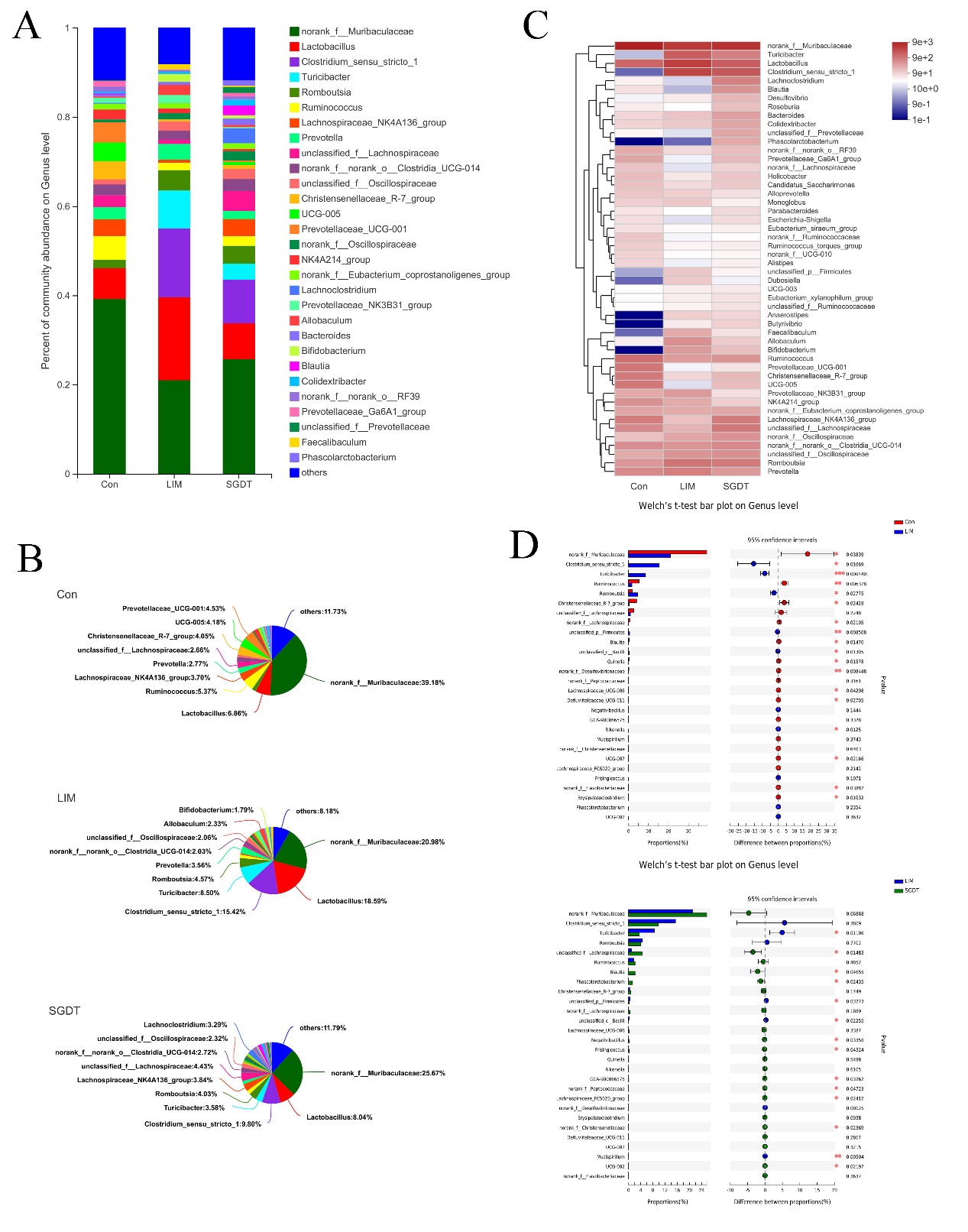


Fig. S10 The effect of SGD on the structure of gut microbiota in rats with CCl_4_-induced liver injury was analyzed by Con, LIM and SGDT at the genus level. (A) Community bar chart. (B) Community pie chart. (C) Community heatmap. (D) Test of inter-group differences based on welch's t-test between Con and LIM and between LIM and SGDT.


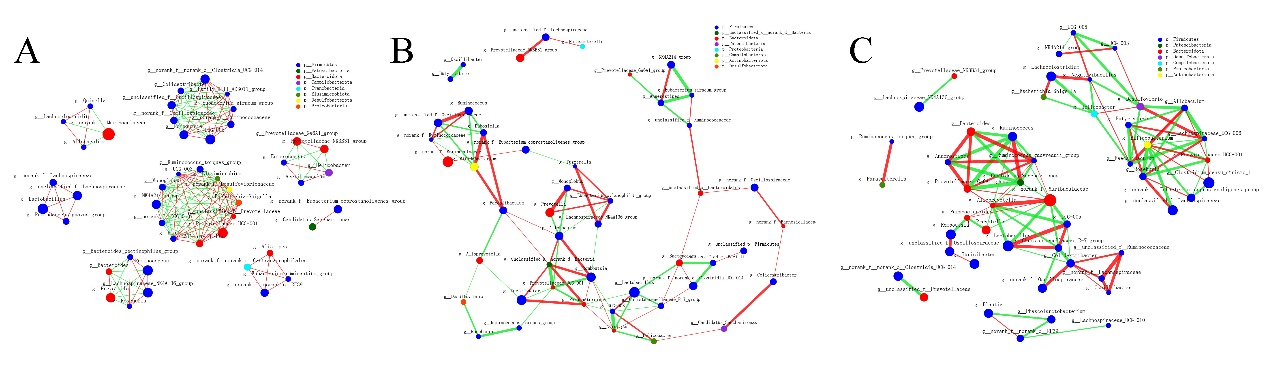


Fig. S11 Correlation network analysis of 50 richest genera in (A) Con, (B) LIM, and (C) SGDT. Analysis by spearman. The color of the nodes in the graph was based on the phylum level, and the size indicated the abundance. The thickness of the line indicated the degree of correlation, red indicated positive, green indicated negative.


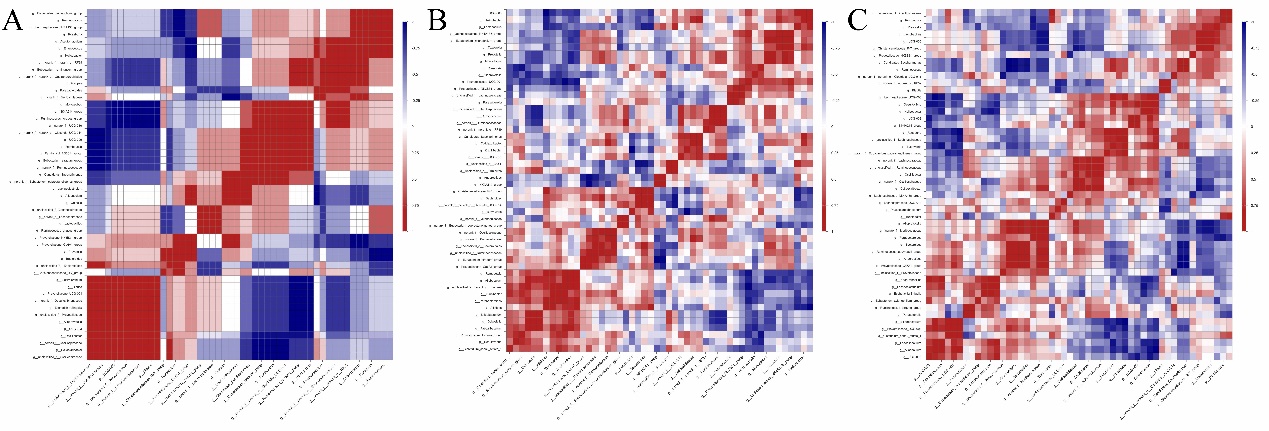


Fig. S12 Heat map for correlation analysis of gut microbiota at genus level. (A) Con. (B) LIM. (C) SGDT. Red indicated positive correlation, blue indicated negative, and darker color indicated higher degree of correlation.


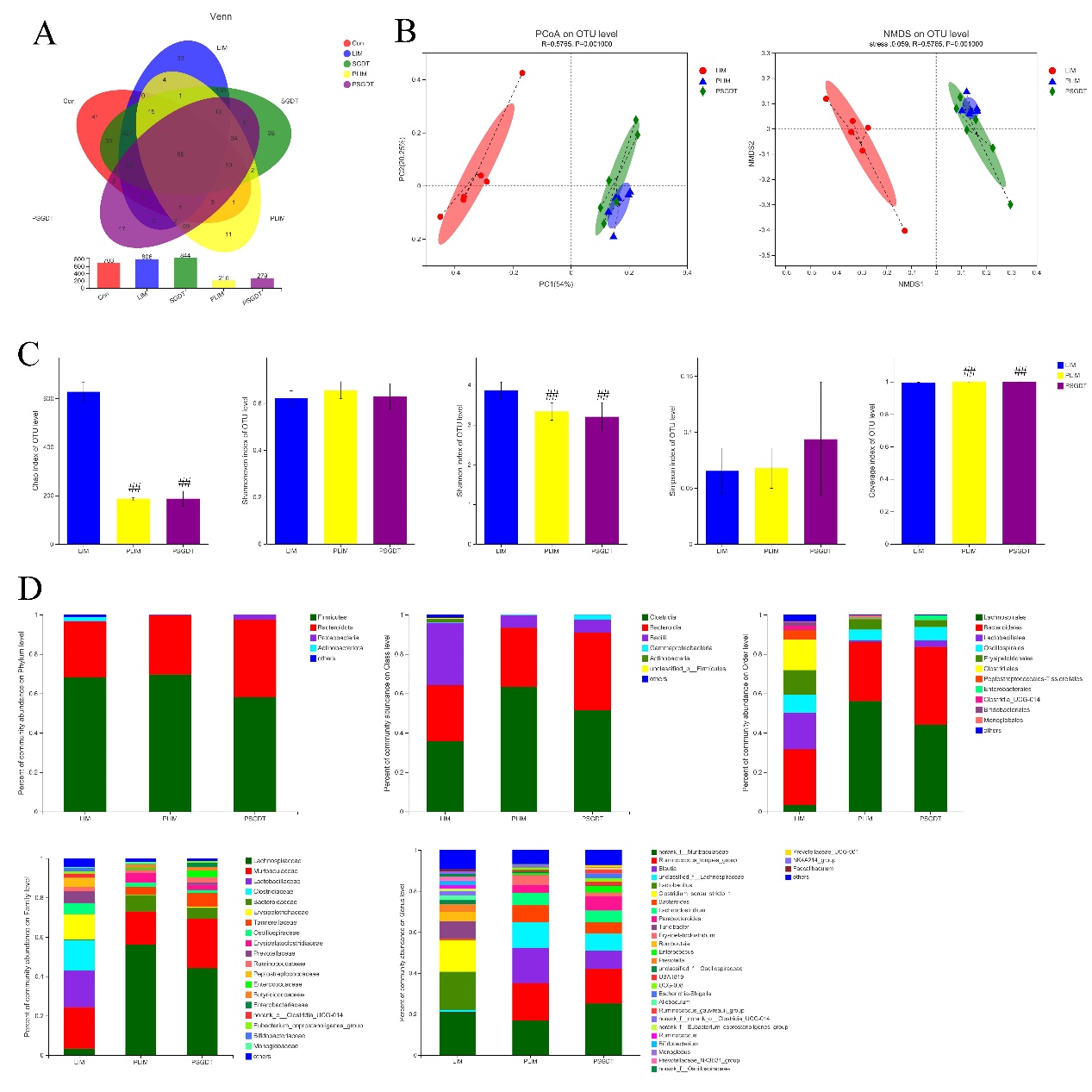


Fig. S13 By analyzing LIM, PLIM and PSGDT, the composition and structure of gut microbiota in PGF rats were compared. (A) Veen plot. (B) PCoA and NMDS analysis. (C) Alpha diversity index, including Chao, Shannoneven, Shannon, Simpson, Coverage. (D) Community bar chart at phylum, class, order, family and genus level.


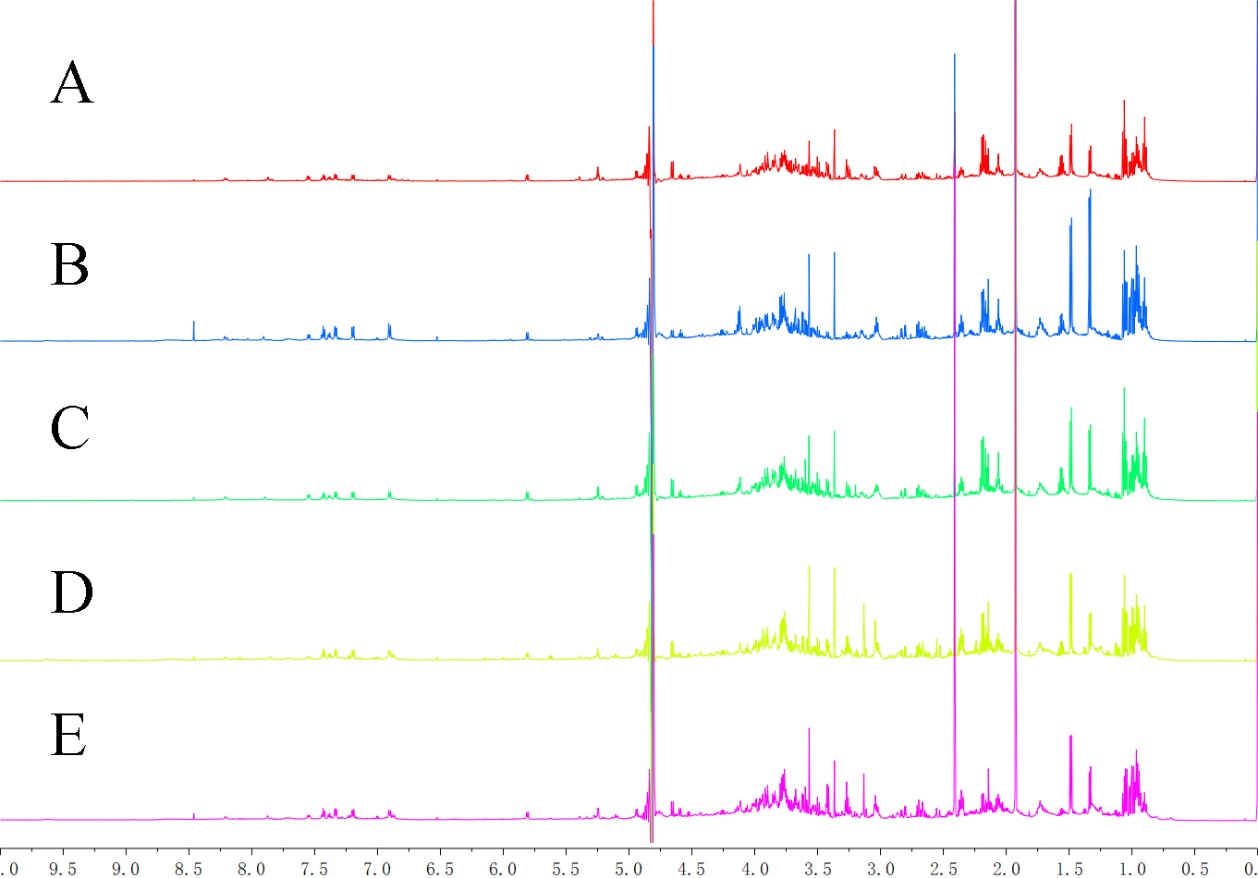


Fig. S14 Representative ^1^H NMR spectra of fecal samples. (A) Con. (B) LIM. (C) SGDT. (D) PLIM. (E) PSGDT.


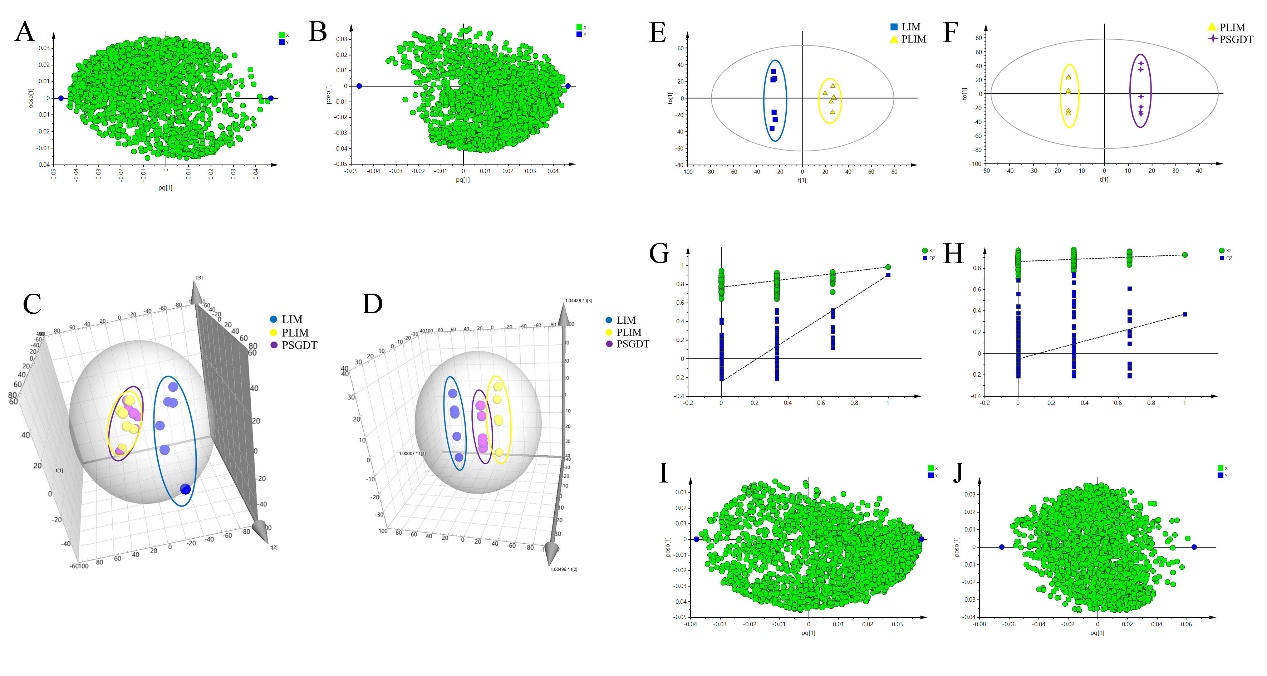


Fig. S15 ^1^H NMR-based loading plots from OPLS-DA between Con and LIM (A), LIM and SGDT (B), and fecal metabolite profiling from PGF rats. (C, D) PCA and OPLS-DA score plots of LIM, PLIM and PSGDT. (E, F) OPLS-DA score plots between LIM and PLIM, PLIM and PSGDT. (G, H) Permutation test plots (200 permutations) between LIM and PLIM (R^2^=0.769, Q^2^=-0.243), PLIM and PSGDT (R^2^=0.864, Q^2^=-0.05). OPLS-DA loading plots between LIM and PLIM (I), PLIM and PSGDT (J).


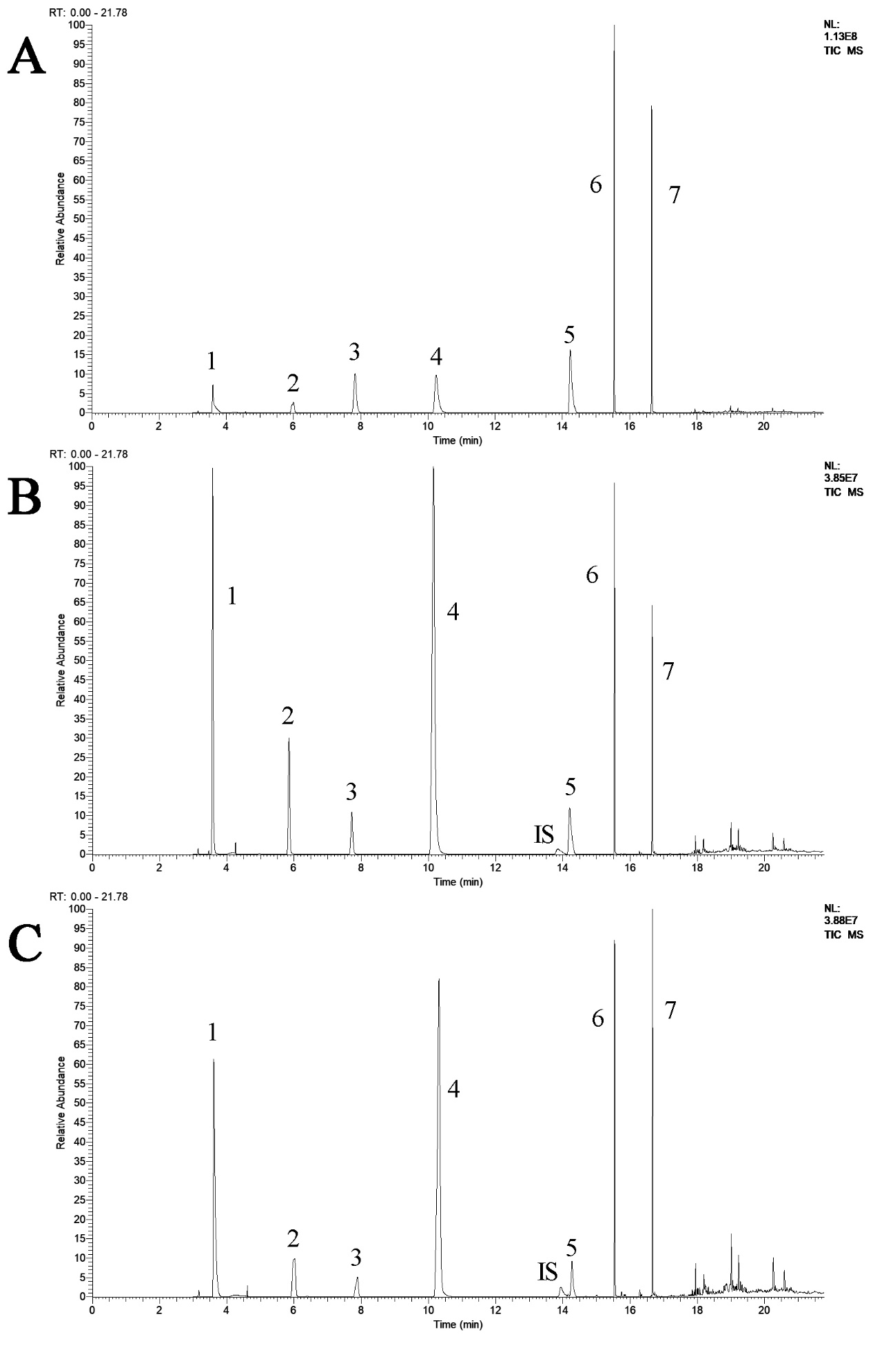


Fig. S16 Typical TICs of SCFAs determined by GC-MS. (A) Standard mixture. (B) Standard mixture containing IS. (C) Samples of cecal contents containing IS. (1) Acetic acid, (2) Propionic acid, (3) Isobutyric acid, (4) Butyric acid, (5) Isovaleric acid, (6) Valeric acid, (7) Caproic acid, (IS) Crotonic acid.


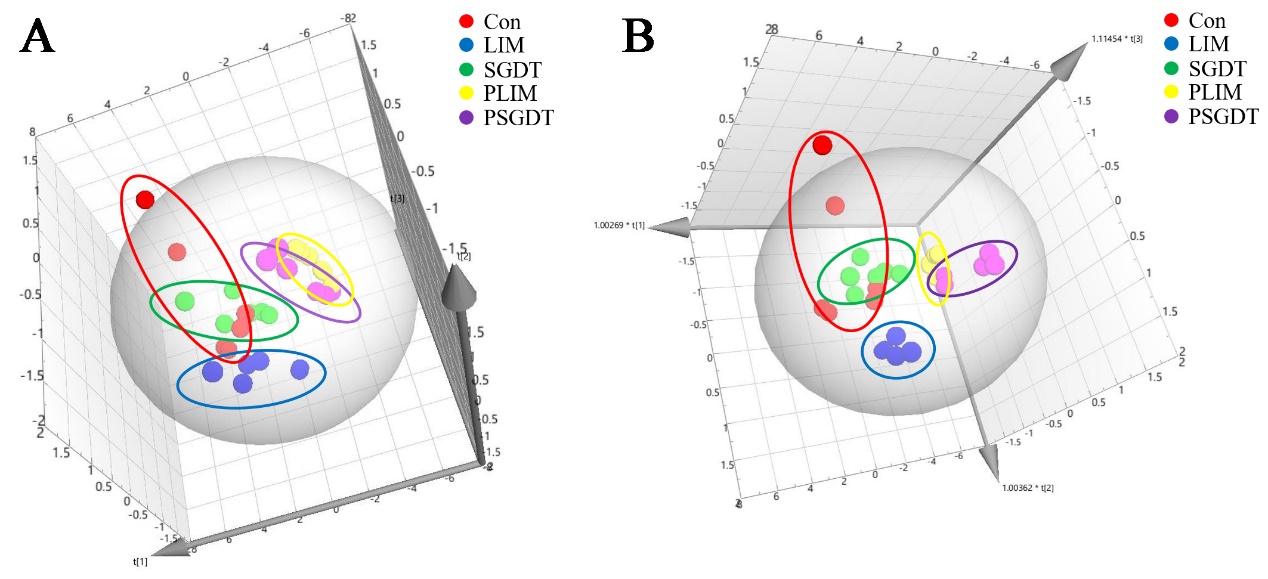


Fig.S17 PCA and OPLS-DA analysis of the effects of SGD treatment on SCFAs in the cecum of rats with liver injury. (A) PCA analysis (R^2^X=0.969, Q^2^=0.843). (B) OPLS-DA analysis (R^2^X=0.982, R^2^Y=0.54, Q^2^=0.307).

**3 Supplementary Tables.**

Table S1 Quantitative ions of derivatives of SCFAs.

| SCFAs | t_R_ (min) | m/z |
| --- | --- | --- |
| Acetic acid | 3.61 | 73 |
| Propionic acid | 6.01 | 87 |
| Isobutyric acid | 7.85 | 89 |
| Butyric acid | 10.28 | 89 |
| Isovaleric acid | 14.25 | 85 |
| Valeric acid | 15.54 | 85 |
| Caproic acid | 16.66 | 99 |
| Crotonic acid | 13.89 | 69 |

Table S2 α-diversity analysis of microbial community structure in five groups

| Group | Richness index | Evenness index | Diversity index | | Coverage index |
| --- | --- | --- | --- | --- | --- |
|  | Chao | Shannoneven | Shannon | Simpson | Coverage |
| Con | 566.95±54.93 | 0.71±0.011 | 4.39±0.12 | 0.038±0.011 | 0.996±0.00044 |
| LIM | 613.73±28.59 | 0.62±0.032^**^ | 3.86±0.20^**^ | 0.066±0.020 | 0.995±0.00049^**^ |
| SGDT | 619.28±39.22 | 0.70±0.038^##^ | 4.35±0.26^##^ | 0.046±0.022 | 0.995±0.00040 |
| PLIM | 186.80±5.25^##^ | 0.65±0.036 | 3.33±0.22^##^ | 0.068±0.018 | 0.999±0.00015^##^ |
| PSGDT | 186.99±30.73^##^ | 0.63±0.054 | 3.20±0.36^##^ | 0.094±0.051 | 0.999±0.00016^##^ |

**p＜0.01, compare with Con; ## p＜0.01, compare with LIM.

Table S3 Genus-level marker bacterial taxa obtained by LEfSe analysis.

| No. | Marker taxa | Con vs LIM | | LIM vs SGDT | |
| --- | --- | --- | --- | --- | --- |
|  |  | Con | LIM | LIM | SGDT |
| 1 | g__Akkermansia |  | √ |  |  |
| 2 | g__Anaerofilum | √ |  |  |  |
| 3 | g__Anaerostipes |  | √ |  |  |
| 4 | g__Bifidobacterium |  | √ |  |  |
| 5 | g__Blautia | √ |  |  | √ |
| 6 | g__Butyrivibrio |  | √ |  |  |
| 7 | g__Candidatus_Stoquefichus |  |  |  | √ |
| 8 | g__Christensenellaceae_R-7_group | √ |  |  |  |
| 9 | g__Clostridium_sensu_stricto_1 |  | √ |  |  |
| 10 | g__Defluviitaleaceae_UCG-011 | √ |  |  |  |
| 11 | g__Dubosiella |  | √ |  |  |
| 12 | g__Erysipelatoclostridium | √ |  |  | √ |
| 13 | g__Erysipelotrichaceae_UCG-003 |  |  |  | √ |
| 14 | g__Escherichia-Shigella |  |  |  | √ |
| 15 | g__Eubacterium_brachy_group | √ |  |  |  |
| 16 | g__Faecalibaculum |  | √ |  |  |
| 17 | g__Family_XIII_AD3011_group | √ |  |  |  |
| 18 | g__Holdemania |  |  |  | √ |
| 19 | g__Lachnoclostridium | √ |  |  | √ |
| 20 | g__Lachnospiraceae_FCS020_group |  |  |  | √ |
| 21 | g__Lachnospiraceae_NK4A136_group | √ |  |  | √ |
| 22 | g__Lachnospiraceae_UCG-006 | √ |  |  |  |
| 23 | g__Mucispirillum |  |  | √ |  |
| 24 | g__Negativibacillus |  |  |  | √ |
| 25 | g__norank_f__Christensenellaceae |  |  |  | √ |
| 26 | g__norank_f__Desulfovibrionaceae | √ |  |  |  |
| 27 | g__norank_f__Flavobacteriaceae | √ |  |  |  |
| 28 | g__norank_f__Lachnospiraceae | √ |  |  | √ |
| 29 | g__norank_f__Muribaculaceae | √ |  |  |  |
| 30 | g__norank_f__norank_o__Coriobacteriales | √ |  |  |  |
| 31 | g__norank_f__Ruminococcaceae | √ |  |  |  |
| 32 | g__Paludicola |  |  |  | √ |
| 33 | g__Parabacteroides |  |  |  | √ |
| 34 | g__Phascolarctobacterium |  |  |  | √ |
| 35 | g__Prevotellaceae_Ga6A1_group |  |  |  | √ |
| 36 | g__Prevotellaceae_UCG-001 | √ |  |  |  |
| 37 | g__Quinella | √ |  |  |  |
| 38 | g__Rikenella |  | √ |  |  |
| 39 | g__Ruminococcus | √ |  |  |  |
| 40 | g__Ruminococcus_gauvreauii_group |  |  |  | √ |
| 41 | g__Ruminococcus_gnavus_group | √ |  |  |  |
| 42 | g__Sellimonas | √ |  |  |  |
| 43 | g__Treponema | √ |  |  |  |
| 44 | g__Turicibacter |  | √ | √ |  |
| 45 | g__UCG-002 |  |  |  | √ |
| 46 | g__UCG-005 | √ |  |  | √ |
| 47 | g__UCG-008 | √ |  |  | √ |
| 48 | g__unclassified_c__Bacilli |  | √ | √ |  |
| 49 | g__unclassified_f__Lachnospiraceae | √ |  |  | √ |
| 50 | g__unclassified_f__Peptostreptococcaceae |  | √ |  |  |
| 51 | g__unclassified_f__Prevotellaceae |  |  |  | √ |
| 52 | g__unclassified_p__Firmicutes |  | √ | √ |  |

√: meaning that the marker bacterial taxa were significantly enriched in the analyses for the different groups (P < 0.05).

Table S4 ^1^H NMR chemical shifts of fecal metabolites.

| No. | Metabolites | Chemical shift (ppm) | No. | Metabolites | Chemical shift (ppm) |
| --- | --- | --- | --- | --- | --- |
| 1 | α-Keto-β-methyl-valerate | 0.888(t), 1.104(d), 2.924(m) | 40 | Taurine | 3.244(t), 3.412(t) |
| 2 | 3-Methyl-2-ketovalerate | 0.888(t), 1.46(m), 2.94(m) | 41 | Trimetlylamine oxide | 3.252(s) |
| 3 | Valine | 0.98(d), 3.588(d) | 42 | Betaine | 3.256(s) |
| 4 | Methylsuccinate | 1.084(d), 2.516(dd) | 43 | Inositol | 3.264(t), 3.524(dd), 3.608(t) |
| 5 | α-Ketoisovalerate | 1.128(d) | 44 | Tryptophan | 3.284(dd), 3.472(dd), |
| 6 | 3-Hydroxybutyric acid | 1.188(d), 2.344(q), 4.132(t) | 45 | Histamine | 3.288(dt) |
| 7 | 2-Hydroxyisobutyrate | 1.264(s),1316(s) | 46 | Proline | 3.348(m), 4.112(m) |
| 8 | Threonine | 1.328(d), 3.58(d) | 47 | α-Glucose | 3.416(m), 3.724(m), 5.216(d) |
| 9 | Lactate | 1.332(d), 4.1(q) | 48 | p-Hydroxyphenylacetate | 3.444(s), 6.864(d) |
| 10 | Cadaverine | 1.46(m),3.012(t) | 49 | Sucrose | 3.46(dd), 4.04(dd) |
| 11 | Ornithine | 1.78(q),3.744(t) | 50 | β-Galactose | 3.496(dd), 3.712(m), 4.592(d) |
| 12 | Isoleucine | 1.96(m), 3.656(d) | 51 | 5-N-acetylneuraminate | 3.504(dd), 3.84(dd) |
| 13 | Methylcysteine | 2.12(s), 3.024(dd), 3.092(dd) | 52 | Phenylacetate | 3.52(s) |
| 14 | Methylcysteine | 2.124(s), 3.952(t) | 53 | Glycine | 3.544(s) |
| 15 | Levulinic acid | 2.208(s), 2.392(t) | 54 | Sarcosine | 3.596(s) |
| 16 | Acetone | 2.228(s) | 55 | α-Galactose | 3.64(dd), 4.584(d), 5.26(d) |
| 17 | 5-Aminovalerate | 2.24(t) | 56 | Uridine | 3.652(d), 3.832(t), 3.96(d) |
| 18 | Ureidopropionate | 2.328(m), 3.152(m) | 57 | Glycerol | 3.68(dd) |
| 19 | Pyruvic acid | 2.364(s) | 58 | Fructose | 3.684(d), 4.024(q), 4.104(s) |
| 20 | Succinate | 2.38(t) | 59 | β-arabinose | 3.692(dd), 3.948(dd) |
| 21 | α-Ketoglutaric acid | 2.428(t) | 60 | Leucine | 3.72(t) |
| 22 | Glutamine | 2.444(m), 3.764(t) | 61 | Lysine | 3.74(t) |
| 23 | Desaminotyrosine | 2.452(t) | 62 | Citrulline | 3.744(t) |
| 24 | Dimethylamine | 2.492(s) | 63 | Cytidine | 3.748(m),3.916(m) |
| 25 | Citrate | 2.576(d) | 64 | Glutamate | 3.752(3.748)(m) |
| 26 | Methionine | 2.624(t), 3.852(m) | 65 | Guanidinoacetic acid | 3.756(s) |
| 27 | Aspartate | 2.708(dd), 3.892(dd) | 66 | N-Acetyl-D-glucosamine | 3.76(m), 5.204(d) |
| 28 | Phenylalanine | 2.848(dd) ,3.272(dd) | 67 | Alanine | 3.768(q) |
| 29 | Trimethylamine | 2.872(s) | 68 | Serine | 3.828(dd), 3.956(dd) |
| 30 | Dimethylglycine | 2.916(s), 3.716(s) | 69 | Creatine | 3.92(s) |
| 31 | Tyramine | 2.932(t), 7.212(d) | 70 | Hippuric Acid | 3.944(d) |
| 32 | Cysteine | 3.048(s), 3.952(s) | 71 | Creatinine | 4.044(s) |
| 33 | Putrescine | 3.048(t) | 72 | Fucose | 5.212(d) |
| 34 | Tyrosine | 3.052(m),3.936(m), 6.88(d), 7.184(d) | 73 | α-arabinose | 5.272(d) |
| 35 | Malonic acid | 3.116(s) | 74 | Uracil | 5.804(d), 7.528(d) |
| 36 | Ethanolamine | 3.128(d), 3.82(d) | 75 | Urocanate | 6.38(d), 7.284(d), 7.816(s) |
| 37 | Choline | 3.196(s), 3.512(t), 4.052(m) | 76 | Adenine | 8.14(s) |
| 38 | Arginine | 3.24(t), 3.764(t) | 77 | Hypoxanthine | 8.192(s), 8.212(s) |
| 39 | Histidine | 3.244(dd), 3.984(t) | 78 | Formate | 8.468(s) |

Table S5 Parameters of the PCA and OPLS-DA models.

| Model | R2X | R2Y | Q2 |
| --- | --- | --- | --- |
| PCA (Con vs LIM vs SGDT) | 0.996 | - | 0.744 |
| OPLS-DA (Con vs LIM vs SGDT) | 0.848 | 0.996 | 0.818 |
| OPLS-DA (Con vs LIM) | 0.842 | 0.999 | 0.899 |
| OPLS-DA (LIM vs SGDT) | 0.812 | 0.999 | 0.88 |
| PCA (LIM vs PLIM vs PSGDT) | 0.836 | - | 0.36 |
| OPLS-DA (LIM vs PLIM vs PSGDT) | 0.814 | 0.996 | 0.584 |
| OPLS-DA (LIM vs PLIM) | 0.62 | 0.994 | 0.928 |
| OPLS-DA (PLIM vs PSGDT) | 0.787 | 1 | 0.552 |

Table S6 Fecal metabolites altered by CCl_4_-induced liver injury or SGD treatment.

| No. | Metabolites | VIP | | *P*-value | | Fold change (Trend) | |
| --- | --- | --- | --- | --- | --- | --- | --- |
|  |  | Con vs LIM | LIM vs SGDT | Con vs LIM | LIM vs SGDT | Con vs LIM | LIM vs SGDT |
| 1 | 5-Aminovalerate | 1.64 | 1.80 | 0.012 | 0.003 | 0.82 (↓^*^) | 1.27 (↑^##^) |
| 2 | 5-N-acetylneuraminate | 2.01 | 2.02 | ＜0.001 | ＜0.001 | 0.69 (↓^**^) | 1.41 (↑^##^) |
| 3 | Acetone | 1.50 | 1.74 | 0.025 | 0.005 | 0.89 (↓^*^) | 1.18 (↑^##^) |
| 4 | Adenine | 1.48 | - | 0.027 | - | 1.54 (↑^*^) | - |
| 5 | Alanine | 1.70 | 1.58 | 0.008 | 0.015 | 0.75 (↓^**^) | 1.19 (↑^#^) |
| 6 | Arginine | 1.67 | 1.60 | 0.009 | 0.014 | 0.67 (↓^**^) | 1.44 (↑^#^) |
| 7 | Aspartate | 1.74 | 2.04 | 0.006 | ＜0.001 | 0.86 (↓^**^) | 1.26 (↑^##^) |
| 8 | Choline | 1.88 | - | 0.002 | - | 0.67 (↓^**^) | - |
| 9 | Citrulline | 2.03 | 2.04 | ＜0.001 | ＜0.001 | 0.67 (↓^**^) | 1.39 (↑^##^) |
| 10 | Creatine | 1.90 | 2.01 | 0.005 | ＜0.001 | 0.69 (↓^**^) | 1.38 (↑^##^) |
| 11 | Creatinine | 1.50 | - | 0.025 | - | 0.76 (↓^*^) | - |
| 12 | Cysteine | 1.48 | - | 0.027 | - | 0.73 (↓^*^) | - |
| 13 | Cytidine | - | 1.81 | - | 0.003 | - | 1.28 (↑^##^) |
| 14 | Dimethylamine | 1.60 | 1.72 | 0.014 | 0.006 | 0.82 (↓^*^) | 1.38 (↑^##^) |
| 15 | Ethanolamine | 1.63 | 1.66 | 0.012 | 0.009 | 0.80 (↓^*^) | 1.19 (↑^##^) |
| 16 | Fructose | 1.82 | 1.75 | 0.003 | 0.005 | 0.76 (↓^**^) | 1.21 (↑^##^) |
| 17 | Fucose | 1.93 | - | 0.004 | - | 0.59 (↓^**^) | - |
| 18 | Glutamate | 1.77 | 1.75 | 0.004 | 0.005 | 0.78 (↓^**^) | 1.24 (↑^##^) |
| 19 | Glutamine | 1.68 | 1.52 | 0.008 | 0.022 | 0.80 (↓^**^) | 1.17 (↑^#^) |
| 20 | Glycerol | 1.80 | 1.97 | 0.009 | 0.001 | 0.72 (↓^**^) | 1.30 (↑^##^) |
| 21 | Glycine | 2.13 | 2.22 | ＜0.001 | ＜0.001 | 0.67 (↓^**^) | 1.54 (↑^##^) |
| 22 | Guanidinoacetic acid | 1.72 | 1.88 | 0.007 | 0.001 | 0.86 (↓^**^) | 1.19 (↑^##^) |
| 23 | Hippuric Acid | 1.74 | 1.89 | 0.006 | 0.001 | 0.72 (↓^**^) | 1.27 (↑^##^) |
| 24 | Inositol | 1.43 | 1.56 | 0.035 | 0.017 | 0.78 (↓^*^) | 1.22 (↑^#^) |
| 25 | Isoleucine | 1.69 | 1.56 | 0.008 | 0.018 | 0.81 (↓^**^) | 1.19 (↑^#^) |
| 26 | Lactate | 1.48 | - | 0.028 | - | 1.70 (↑^*^) | - |
| 27 | Leucine | 1.74 | 1.78 | 0.006 | 0.004 | 0.75 (↓^**^) | 1.22 (↑^##^) |
| 28 | Lysine | 1.82 | 2.11 | 0.003 | ＜0.001 | 0.82 (↓^**^) | 1.26 (↑^##^) |
| 29 | Methionine | 1.80 | - | 0.004 | - | 0.82 (↓^**^) | - |
| 30 | Methylsuccinate | 1.44 | 2.19 | 0.033 | ＜0.001 | 0.88 (↓^*^) | 1.41 (↑^##^) |
| 31 | N-Acetyl-D-glucosamine | 1.41 | - | 0.039 | - | 0.85 (↓^*^) | - |
| 32 | Phenylacetate | 2.06 | 1.93 | ＜0.001 | 0.001 | 0.49 (↓^**^) | 1.59 (↑^##^) |
| 33 | Phenylalanine | - | 1.54 | - | 0.020 | - | 1.49 (↑^#^) |
| 34 | p-Hydroxyphenylacetate | 2.01 | - | ＜0.001 | - | 0.52 (↓^**^) | - |
| 35 | Proline | 1.39 | 1.94 | 0.042 | 0.001 | 0.87 (↓^*^) | 1.31 (↑^##^) |
| 36 | Putrescine | 1.74 | - | 0.006 | - | 0.76 (↓^**^) | - |
| 37 | Pyruvic acid | - | 1.84 | - | 0.006 | - | 1.40 (↑^##^) |
| 38 | Sarcosine | 1.75 | - | 0.005 | - | 0.71 (↓^**^) | - |
| 39 | Serine | 1.77 | - | 0.004 | - | 0.77 (↓^**^) | - |
| 40 | Succinate | 1.96 | - | 0.001 | - | 1.16 (↑^**^) | - |
| 41 | Sucrose | 1.99 | - | ＜0.001 | - | 0.55 (↓^**^) | - |
| 42 | Taurine | 1.88 | 1.76 | 0.002 | 0.004 | 0.57 (↓^**^) | 1.63 (↑^##^) |
| 43 | Threonine | 1.97 | 1.59 | 0.002 | 0.014 | 1.59 (↑^**^) | 0.76 (↓^#^) |
| 44 | Trimetlylamine oxide | 1.36 | 1.53 | 0.049 | 0.021 | 0.76 (↓^*^) | 1.34 (↑^#^) |
| 45 | Tryptophan | 2.03 | 1.94 | ＜0.001 | 0.001 | 0.57 (^*^↓^*^) | 1.59 (↑^##^) |
| 46 | Tyrosine | 1.63 | 1.80 | 0.012 | 0.003 | 0.85 (↓^*^) | 1.17 (↑^##^) |
| 47 | Uridine | 1.77 | 2.12 | 0.005 | ＜0.001 | 0.80 (↓^**^) | 1.33 (↑^##^) |
| 48 | Valine | - | 1.76 | - | 0.004 | - | 1.34 (↑^##^) |
| 49 | α-Arabinose | 2.04 | 1.57 | ＜0.001 | 0.016 | 0.58 (↓^**^) | 1.24 (↑^#^) |
| 50 | α-Galactose | 1.68 | 1.38 | 0.009 | 0.042 | 0.76 (↓^**^) | 1.18 (↑^#^) |
| 51 | α-Glucose | 2.04 | 1.97 | ＜0.001 | 0.001 | 0.59 (↓^**^) | 1.49 (↑^##^) |
| 52 | α-Ketoglutaric acid | 1.56 | 1.90 | 0.018 | 0.001 | 0.83 (↓^*^) | 1.19 (↑^##^) |
| 53 | α-Ketoisovalerate | 1.54 | 1.84 | 0.021 | 0.002 | 0.81 (↓^*^) | 1.32 (↑^##^) |
| 54 | β-arabinose | 1.87 | 2.05 | 0.002 | ＜0.001 | 0.71 (↓^**^) | 1.41 (↑^##^) |
| 55 | β-Galactose | 2.01 | 2.13 | ＜0.001 | ＜0.001 | 0.64 (↓^**^) | 1.47 (↑^##^) |

Fold change was calculated from the integral area of the corresponding chemical shift. “↑” and “↓” means the metabolite was up- and down-regulated, “-”means the metabolite do not produce significant change. *p＜0.05, **p＜0.01, compare with Con; # p＜0.05, ## p＜0.01, compare with LIM.

Table S7 Fecal metabolites altered by CCl_4_-induced liver injury or SGD treatment.

| No. | Metabolites | VIP | | *P*-value | | | Fold change (Trend) | | |
| --- | --- | --- | --- | --- | --- | --- | --- | --- | --- |
|  |  | LIM vs PLIM | PLIM vs PSGDT | LIM vs PLIM | PLIM vs PSGDT | LIM vs PLIM | | PLIM vs PSGDT | |
| 1 | 3-Hydroxybutyric acid | 1.23 | - | 0.037 | - | | 0.62 (↓^#^) | | - |
| 2 | 3-Methyl-2-ketovalerate | 1.28 | - | 0.027 | - | | 0.86 (↓^#^) | | - |
| 3 | Betaine | - | 2.30 | - | 0.019 | | - | | 0.80 (↓^^^) |
| 4 | Citrate | 1.41 | - | 0.006 | - | | 1.37 (↑^##^) | | - |
| 5 | Desaminotyrosine | - | 2.54 | - | 0.003 | | - | | 1.15 (↑^^^^) |
| 6 | Dimethylglycine | 1.23 | - | 0.023 | - | | 1.23 (↑^#^) | | - |
| 7 | Formate | 1.83 | - | ＜0.001 | - | | 0.29 (↓^##^) | | - |
| 8 | Histamine | - | 2.67 | - | 0.001 | | - | | 0.73 (↓^^^^) |
| 9 | Hypoxanthine | 1.23 | - | 0.024 | - | | 1.39 (↑^#^) | | - |
| 10 | Levulinic acid | - | 2.02 | - | 0.032 | | - | | 0.90 (↓^^^) |
| 11 | Methylcysteine | 1.22 | - | 0.025 | - | | 1.26 (↑^#^) | | - |
| 12 | Methylsuccinate | - | 2.44 | - | 0.005 | | - | | 1.16 (↑^^^^) |
| 13 | Succinate | - | 1.90 | - | 0.047 | | - | | 0.85 (↓^^^) |
| 14 | Trimethylamine | 1.29 | - | 0.031 | - | | 2.67 (↑^#^) | | - |
| 15 | Tyramine | - | 2.56 | - | 0.003 | | - | | 1.47 (↑^^^^) |
| 16 | Uracil | 1.34 | - | 0.011 | - | | 1.34 (↑^#^) | | - |
| 17 | Ureidopropionate | 1.68 | 2.24 | ＜0.001 | 0.014 | | 1.35 (↑^##^) | | 0.86 (↓^^^) |
| 18 | Urocanate | 1.17 | - | 0.034 | - | | 1.22 (↑^#^) | | - |
| 19 | α-Ketoglutaric acid | - | 2.02 | - | 0.031 | | - | | 1.22 (↑^^^) |
| 20 | α-Keto-β-methyl-valerate | 1.27 | - | 0.035 | - | | 0.62 (↓^#^) | | - |

Fold change was calculated from the integral area of the corresponding chemical shift. “↑” and “↓” means the metabolite was up- and down-regulated, “-”means the metabolite do not produce significant change. # p＜0.05, ## p＜0.01, compare with LIM; ^p＜0.05, ^^p＜0.01, compare with PLIM.

Table S8 Regression equation, linear range and LLOQ for SCFAs.

| SCFAs | t_R_ (min) | Calibration equation | R^2^ | Linear range（μg/mL） | LLOQ（μg/mL） |
| --- | --- | --- | --- | --- | --- |
| Acetic acid | 3.61 | y=0.0424 x-0.2302 | 0.9970 | 1-500 | 1 |
| Propionic acid | 6.01 | y=0.0182 x+0.3539 | 0.9979 | 1-500 | 1 |
| Isobutyric acid | 7.85 | y=0.0806 x+0.0070 | 0.9985 | 0.1-50 | 0.1 |
| Butyric acid | 10.28 | y=0.1121 x-0.2691 | 0.9996 | 1-500 | 1 |
| Isovaleric acid | 14.25 | y=0.1297 x-0.0196 | 0.9996 | 0.1-50 | 0.1 |
| Valeric acid | 15.54 | y=0.1741 x-0.0673 | 0.9990 | 0.1-50 | 0.1 |
| Caproic acid | 16.66 | y=0.1124 x-0.0262 | 0.9995 | 0.1-50 | 0.1 |

Table S9 Precision, accuracy, recovery and matrix effect of SCFAs.

| SCFAs | Concentration (μg/ml) | Intra-day RSD (%) | Inter-day RSD (%) | Accuracy RE (%) | Recovery  (%, mean ± SD) | Matrix effect  (%, mean ± SD) |
| --- | --- | --- | --- | --- | --- | --- |
| Acetic acid | 2 | 8.4 | 7.8 | 5.6 | 87.4 ± 1.6 | 92.1 ± 1.9 |
|  | 40 | 1.2 | 3.7 | 4.5 | 97.3 ± 2.4 | 94.8 ± 4.7 |
|  | 400 | 9.4 | 4.5 | 5.8 | 94.7 ± 4.3 | 97.4 ± 5.1 |
| Propionic acid | 2 | 4.1 | 3.4 | -1.8 | 96.1 ± 4.2 | 97.7 ± 1.2 |
|  | 40 | 2.1 | 3.4 | 9.3 | 94.4 ± 3.1 | 95.5 ± 3.3 |
|  | 400 | 4.3 | 6.5 | -3.3 | 93.2 ± 5.1 | 96.5 ± 2.8 |
| Isobutyric acid | 0.2 | 7.3 | 6.3 | 10.2 | 96.7 ± 3.9 | 96.5 ± 5.5 |
|  | 4 | 1.3 | 4.4 | 4.6 | 95.5 ± 3.7 | 91.2 ± 5.9 |
|  | 40 | 8.7 | 11.4 | 6.1 | 96.4 ± 1.4 | 94.8 ± 3.7 |
| Butyric acid | 2 | 1.3 | 3.8 | 8.1 | 90.6 ± 5.1 | 91.2 ± 5.2 |
|  | 40 | 9.5 | 7.3 | -7.6 | 97.2 ± 2.4 | 96.6 ± 4.7 |
|  | 400 | 8.1 | 5.2 | 8.1 | 87.9 ± 4.4 | 93.7 ± 1.4 |
| Isovaleric acid | 0.2 | 9.2 | 4.8 | 2.7 | 90.6 ± 1.4 | 90.3 ± 2.8 |
|  | 4 | 11.3 | 6.4 | 4.9 | 90.7 ± 4.3 | 96.9 ± 2.6 |
|  | 40 | 8.6 | 9.6 | 3.7 | 86.7 ± 1.3 | 93.2 ± 4.8 |
| Valeric acid | 0.2 | 5.7 | 7.3 | 9.7 | 88.3 ± 5.7 | 96.3 ± 1.2 |
|  | 4 | 2.8 | 2.6 | 1.1 | 91.7 ± 4.4 | 96.7 ± 5.4 |
|  | 40 | 4.4 | 7.9 | 2.1 | 90.8 ± 2.9 | 97.2 ± 4.5 |
| Caproic acid | 0.2 | 8.4 | 7.8 | 5.6 | 87.4 ± 1.5 | 92.1 ± 2.9 |
|  | 4 | 1.2 | 3.7 | -4.5 | 97.3 ± 2.3 | 94.3 ± 1.1 |
|  | 40 | 9.4 | 4.5 | 5.8 | 94.7 ± 2.8 | 97.4 ± 3.9 |

Table. S10 Stability of SCFAs.

| Analytes | Concentration (ng/ml) | 4°C for 24 h | | Three freeze-thaw cycles | | −80 °C for 30 days | |
| --- | --- | --- | --- | --- | --- | --- | --- |
|  |  | RE (%) | RSD (%) | RE (%) | RSD (%) | RE (%) | RSD (%) |
| Acetic acid | 2 | 2.7 | 2.8 | 3.2 | 3.7 | -5.2 | 6.2 |
|  | 40 | 7.2 | 5.6 | 4.5 | 3.4 | 5.6 | 1.8 |
|  | 400 | -2.9 | 2.3 | -5.6 | 4.4 | 7.7 | 1.8 |
| Propionic acid | 2 | 4.8 | 8.3 | -3.1 | 6.9 | -8.3 | 5.7 |
|  | 40 | 3.8 | 7.8 | 2.5 | 6.5 | -8.9 | 5.6 |
|  | 400 | -2.5 | 6.9 | 2.2 | 8.5 | 2.4 | 5.5 |
| Isobutyric acid | 0.2 | 2.9 | 6.9 | -4.3 | 3.4 | 2.4 | 6.5 |
|  | 4 | 8.8 | 7.1 | 7.1 | 3.4 | -8.9 | 6.1 |
|  | 40 | 7.3 | 8.5 | 6.3 | 2.5 | 5.9 | 3.8 |
| Butyric acid | 2 | -2.1 | 4.6 | 4.1 | 3.7 | 7.5 | 3.4 |
|  | 40 | 2.4 | 8.1 | 5.4 | 7.5 | -2.2 | 4.3 |
|  | 400 | -2.9 | 2.5 | -5.5 | 3.6 | -5.8 | 7.2 |
| Isovaleric acid | 0.2 | -5.9 | 2.2 | -4.8 | 4.6 | 8.2 | 4.4 |
|  | 4 | 2.3 | 3.2 | -3.8 | 4.3 | -4.4 | 4.4 |
|  | 40 | -2.5 | 6.4 | 8.2 | 4.5 | 4.3 | 4.3 |
| Valeric acid | 0.2 | -2.7 | 5.2 | 5.9 | 6.4 | 8.3 | 4.8 |
|  | 4 | -7.5 | 2.8 | -2.3 | 5.7 | 4.3 | 2.6 |
|  | 40 | 4.6 | 6.5 | 5.4 | 5.1 | 7.1 | 8.1 |
| Caproic acid | 0.2 | 2.7 | 2.8 | -3.2 | 3.7 | -5.2 | 6.2 |
|  | 4 | -7.2 | 5.6 | -4.7 | 3.4 | -5.6 | 1.8 |
|  | 40 | 2.9 | 2.3 | 5.6 | 4.4 | -7.7 | 1.8 |
